# Evaluation of core genome and whole genome multilocus sequence typing schemes for *Campylobacter jejuni* and *Campylobacter coli* outbreak detection in the USA

**DOI:** 10.1099/mgen.0.001012

**Published:** 2023-05-03

**Authors:** Lavin A. Joseph, Taylor Griswold, Eshaw Vidyaprakash, Sung B. Im, Grant M. Williams, Hannes A. Pouseele, Kelley B. Hise, Heather A. Carleton

**Affiliations:** ^1^​ Division of Foodborne, Waterborne, and Environmental Diseases, Centers for Disease Control and Prevention, Atlanta, GA, USA; ^2^​ bioMérieux SA, Sint-Martens-Latem, Belgium

**Keywords:** *Campylobacter*, cgMLST, wgMLST, hqSNP, outbreak

## Abstract

*
Campylobacter* is a leading causing of bacterial foodborne and zoonotic illnesses in the USA. Pulsed-field gene electrophoresis (PFGE) and 7-gene multilocus sequence typing (MLST) have been historically used to differentiate sporadic from outbreak *Campylobacter* isolates. Whole genome sequencing (WGS) has been shown to provide superior resolution and concordance with epidemiological data when compared with PFGE and 7-gene MLST during outbreak investigations. In this study, we evaluated epidemiological concordance for high-quality SNP (hqSNP), core genome (cg)MLST and whole genome (wg)MLST to cluster or differentiate outbreak-associated and sporadic *Campylobacter jejuni* and *Campylobacter coli* isolates. Phylogenetic hqSNP, cgMLST and wgMLST analyses were also compared using Baker’s gamma index (BGI) and cophenetic correlation coefficients. Pairwise distances comparing all three analysis methods were compared using linear regression models. Our results showed that 68/73 sporadic *C. jejuni* and *C. coli* isolates were differentiated from outbreak-associated isolates using all three methods. There was a high correlation between cgMLST and wgMLST analyses of the isolates; the BGI, cophenetic correlation coefficient, linear regression model *R* ^2^ and Pearson correlation coefficients were >0.90. The correlation was sometimes lower comparing hqSNP analysis to the MLST-based methods; the linear regression model *R* ^2^ and Pearson correlation coefficients were between 0.60 and 0.86, and the BGI and cophenetic correlation coefficient were between 0.63 and 0.86 for some outbreak isolates. We demonstrated that *C. jejuni* and *C. coli* isolates clustered in concordance with epidemiological data using WGS-based analysis methods. Discrepancies between allele and SNP-based approaches may reflect the differences between how genomic variation (SNPs and indels) are captured between the two methods. Since cgMLST examines allele differences in genes that are common in most isolates being compared, it is well suited to surveillance: searching large genomic databases for similar isolates is easily and efficiently done using allelic profiles. On the other hand, use of an hqSNP approach is much more computer intensive and not scalable to large sets of genomes. If further resolution between potential outbreak isolates is needed, wgMLST or hqSNP analysis can be used.

## Data Summary

Raw sequence data files (Illumina reads) for all 315 isolates have been deposited in SRA under Bioproject PRJNA239251. The list of Biosample and SRA accession numbers for these samples is presented in Table S1 (available with the online version of this article). The list of Biosample and SRR accession numbers for additional closed reference sequences used in hqSNP analysis is given in Table 2.

**Impact Statement**

*
Campylobacter* is a leading cause of foodborne and zoonotic illnesses in the USA. PulseNet, the national molecular subtyping network in the USA that detects potential outbreak clusters of foodborne, waterborne and zoonotic cases, uses whole genome sequencing (WGS) for identification of clusters. We compared the ability of different WGS analysis methods to cluster epidemiologically linked *Campylobacter* isolates and to separate non-epidemiologically linked sporadic isolates that were indistinguishable by 7-gene multilocus sequence typing (7-gene MLST) and PFGE. Our results showed that core genome (cg)MLST, whole genome (wg)MLST and high-quality SNP (hqSNP) analysis results were highly concordant with available epidemiology information and clustered the *Campylobacter* isolates similarly. A stable isolate allele profile is generated by cg/wgMLST analyses, enabling rapid comparisons between isolates during an outbreak investigation, while for hqSNP analyses part, if not all, of the analysis has to be repeated to compare additional isolates. Therefore, cg/wgMLST may be better adapted for public health surveillance. Furthermore, we found that cgMLST is sufficient for *Campylobacter jejuni* and *Campylobacter coli* outbreak detection and surveillance while wgMLST can be used to further differentiate isolates if needed.

## Introduction

*Campylobacter* is a leading cause of bacterial foodborne and zoonotic illness in the USA, resulting in an estimated 1.5 million illnesses, 19 300 hospitalizations and 240 deaths annually [1]. *Campylobacter* infections may result in acute gastroenteritis with diarrhoea, nausea, abdominal pain, fever and/or vomiting [2]. In most cases, the patients recover completely; however, some patients may have long-term complications such as reactive arthritis, inflammatory bowel syndrome or Guillain–Barré syndrome [2–5]. Even though most *Campylobacter* cases are sporadic, previous foodborne and zoonotic outbreaks have been linked to consumption of raw milk, contaminated water, chicken meat, raw peas, and exposure to dogs [6–14].

PFGE and 7-gene multilocus sequence typing (7-gene MLST) have been used historically to differentiate sporadic from outbreak-related *Campylobacter* isolates [7, 8, 15, 16]. However, whole genome sequencing (WGS) has been shown to provide superior resolution and concordance with epidemiological data when compared with PFGE and 7-gene MLST during foodborne outbreak investigations [14, 17–21]. WGS data are also more comprehensive by providing information about the entire genome compared to PFGE and 7-gene MLST, which target limited regions of the genome [22–24]. There are three main approaches that can be employed to analyse WGS data for *Campylobacter* surveillance and outbreak investigations: (i) high-quality SNP (hqSNP) analysis, which compares isolate genomes to a closely related reference sequence to derive SNP differences; (ii) core genome (cg)MLST analysis, which examines differences in the loci found in >95 % of the strains of the reference organisms used to build the allele scheme; and (iii) whole genome (wg)MLST analysis, which examines differences in all loci found in the reference strains used to build the allele schemes [14, 18, 24, 25].

Public health microbiologists and bioinformaticians typically use WGS data to detect outbreaks by identifying sequences that are within 10 SNPs or allele differences of each other [26–30]. These outbreaks are likely to be caused by consumption of a single food source during a temporarily or geographically localized event and are termed clonal outbreaks [31–34]. The food sources may also be contaminated with multiple strains, leading to polyclonal or multi-species outbreaks [34]. In addition, outbreaks can also have a zoonotic or an environmental source, resulting in an outbreak with greater genomic diversity compared to clonal outbreaks [13, 14, 35].

PulseNet, the national laboratory network in the USA that connects foodborne-, waterborne- and One Health-related outbreak-causing illnesses, uses cgMLST and wgMLST schemes to subtype foodborne pathogens, such as *Campylobacter jejuni* and *Campylobacter coli* [30]. The cgMLST scheme (1 343 loci) was developed by our colleagues at the University of Oxford, UK, and is available at http://pubmlst.org/campylobacter [26]. In addition, CDC has developed an hqSNP pipeline [36], which has been used in previous outbreak investigations [14, 37]. However, hqSNP analysis is dependent on a priori knowledge of isolates to select an appropriate reference genome, which is not required for sequences analysed using the cgMLST and wgMLST schemes [38, 39].

In this study, we examined the ability of three WGS-based subtyping methods (wgMLST, cgMLST and hqSNP) to identify epidemiologically associated isolates in *C. jejuni* and *C. coli* outbreaks and differentiate each from sporadic isolates. In addition, we compared the phylogenetic topologies of the three WGS-based subtyping methods and the genetic pairwise distances of the foodborne or zoonotic outbreak-related isolate sequences. Finally, we assessed the effects of sequence quality on cgMLST and wgMLST allele calls.

## Methods

### Isolates and whole genome sequencing

A total of 237 *C *. *jejuni* and five *C *. *coli* isolates from 16 foodborne and zoonotic outbreaks and 73 sporadic isolates were selected for this study (Table S1). The 16 outbreaks were identified between 2008 and 2021 and included epidemiologically linked cases with confirmed sources of illness. The sporadic isolates, 69 *C *. *jejuni* and four *C *. *coli *, were chosen based on indistinguishable PFGE pattern combinations (*Sma*I/*Kpn*I) and/or 7-gene MLST sequence types (STs) when compared to the corresponding outbreak isolates, except in cases where there was only one isolate in cgMLST clades as shown in Table S1. The sporadic isolates were recovered within the same year as the outbreak isolates except for five outbreaks where sporadic isolates from other years were used because an insufficient number were available from the outbreak year. There were no obvious epidemiological connections between the sporadic and outbreak isolates. Sporadic isolates corresponding to outbreak 1008MTDBR-1 were not available to be included in the analysis (Table S1); there were no sporadic isolates matching by PFGE and/or 7-gene MLST ST in the PulseNet *Campylobacter* National Database. PFGE and 7-gene MLST were performed as described below. Isolate information is listed in Table S1. The confirmed source of each outbreak is listed in Table 1. The outbreak codes listed in Table 1 were generated as previously described [30, 40]. Outbreak clades from polyclonal outbreaks were delimited based on the cgMLST phylogenetic tree topology and were designated with letters (i.e. a, b, c). WGS was performed on Illumina sequencers, using the Illumina Nextera XT or DNA Prep library preparation kits, by public health laboratories (PHLs) or CDC according to WGS protocols listed online: https://www.aphl.org/programs/food_safety/Pages/PulseNet-International-SOPs.aspx.

**Table 1.** Ranges of pairwise hqSNP and allele differences within outbreaks/outbreak clades and including sporadic isolates

Outbreak or clades*	No. of isolates	Range of pairwise hqSNP and allele differences (outbreak isolates)			Outbreak source	Range of hqSNP and allele differences (including sporadic isolates)		
		hqSNP	cgMLST	wgMLST		hqSNP	cgMLST	wgMLST
0810PADBR-1	15	0–1	0	0–4	Raw milk	102–149	12–30	17–38
1008MTDBR-1a	15	0–3	0–3	0–3	Water	†	†	†
1302AKDBB-1	5	0–2	0	0–1	Raw milk	123–343	69–119	93–151
1506UTDBR-1a	2	19	12	17	Irrigation water	85–2597	34–563	42–624
1506UTDBR-1b	8	0–3	0–2	0–2		77–80	47–79	83–86
1509VTDBR-1	6	0–5	0–10	0–12	Poultry	19–225	5–131	31–144
1510WIDBR-1	10	0–3	0–2	0–2	Church fundraiser	20–454	7–95	11–106
1602VTDBR-1	4	0–1	0	0–2	Chicken liver	31–294	16–107	20–125
1607CADBR-1a	2	0	0	2	Mexican taqueria	250	150–151	163–166
1607CADBR-1b	2	0	0	0		926–928	339–340	383–384
1607CADBR-1c	5	0	0	0		92–335	49–256	54–294
1612OHDBR-1	3	0	0–1	0–1	Raw milk	262–424	80–123	91–142
1707MADBR-1a	3	10–27	5–8	9–14	Chicken liver	56–504	14–105	25–129
1707MADBR-1b	2	0	0	0		165–409	39–87	53–106
1707MADBR-1c	2	1	1	1		278–535	71–123	90–147
1708FLDBR-1a	22	0–163	0–40	0–58	Pet store puppies	453–1115	174–315	193–366
1708FLDBR-1b	40	0–39	0–28	0–31		58–698	34–225	36–250
1802VADBR-1	3	0	0–1	0–1	Chicken liver	12–797	6–258	20–309
1906NVDBR-1a	21	0–104	0–48	0–58	Pet store puppies	68–338	54–129	62–135
1906NVDBR-1b	11	0–2	0–5	0–6		550–775	226–262	253–289
1906NVDBR-1c	25	0–124	0–59	0–78		209–386	108–138	122–165
1907VTDBR-1	5	0–1	0–1	0–2	Poultry	7–816	4–237	5–292
2102OHDBR-1	11	0–1	0	0–1	Raw milk	133–438	53–107	57–124

Outbreak clades were delimited from polyclonal outbreaks based on the cgMLST phylogenetic tree topology designated with letters (i.e. a, b, c).

Sporadic isolates were not chosen for singletons in each outbreak.

*1008MTDBR-1 clades b–f and 1607CADBR-1 clades d and e are not shown because only one isolate represented each clade.

†This outbreak did not have corresponding sporadic isolates; there were no PFGE or 7-gene MLST matching non-outbreak isolates in the *Campylobacter* National Database.

Total number of cgMLST loci: 1343; total number of cgMLST loci + accessory genome loci: 6623.

### Pulsed-field gel electrophoresis

PFGE was performed by PHLs or CDC on a subset of isolates using the standard operating procedure for PulseNet PFGE of *C. jejuni* [22]. PFGE patterns (*Sma*I only; or *Sma*I and *Kpn*I) were analysed in BioNumerics v6.6.10 (bioMérieux), uploaded to the PulseNet *Campylobacter* National Database, and designated according to the PulseNet naming convention [30, 40].

### Whole genome sequencing analysis

Illumina sequence read files for all outbreak and sporadic isolates were linked to BioNumerics v7.6.3 (bioMérieux). *De novo* assembly of sequences was performed using SPAdes v3.7.1 (minimum coverage=5×, expected coverage=20×, minimum contig length=500, low-coverage filtering threshold=10 %). Consensus allele calls were generated from the combination of assembly-free and assembly-based allele calling. Assembly-free allele calls were determined using the following settings: kmer size=35 bp, minimum coverage=3×, minimum forward=1× and minimum reverse=1×. Assembly-based allele calls were determined using the following settings: minimum similarity and minimum homology=70 %, start/stop codon present and <100 bp insertions/deletions. Reference allele sequences used to call new alleles per locus are in the *Campylobacter* wgMLST Reference Alleles Supplementary data file.

### Sequence quality assessment

**Fig. 1.**
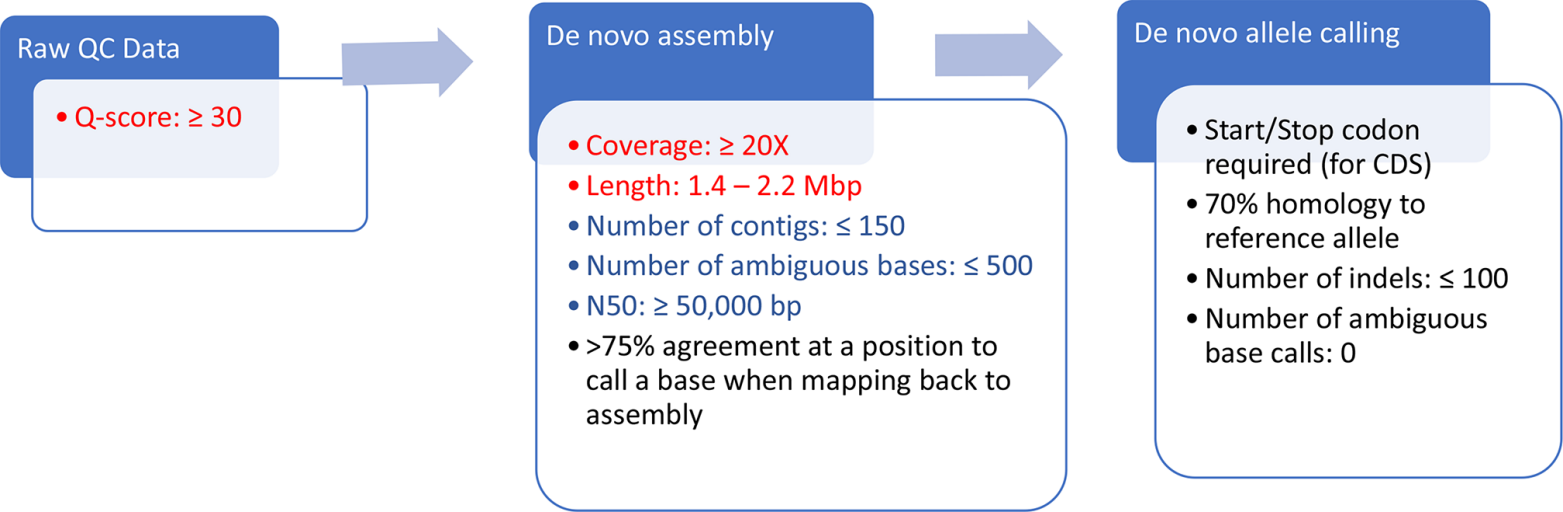
PulseNet *Campylobacter* allele calling workflow. Sequences must pass the quality thresholds in red, otherwise they are rejected. The metrics in blue are evaluated for quality assessment of sequences but are not used to reject sequences. The parameters in black are settings used in BioNumerics. CDS, coding sequence.

PulseNet quality control requirements for *Campylobacter* include Q-score ≥30, 1.4–2.2 Mb sequence length, ≥20× average *de novo* coverage and ≥85 % of core genome loci with allele calls [14]. Sequences used in this analysis, which had Q-scores ≥32, had sequence lengths ranging from 1.59 to 2.12 Mb, had average *de novo* coverages ≥29× and had allele calls (range 1 142–1 330) present for 85–99 % of core genome loci, met the required criteria for PulseNet quality control thresholds and were uploaded to the PulseNet *Campylobacter* National Database. The *Campylobacter* allele calling workflow is illustrated in Fig. 1. Additional sequences from epidemiologically unrelated isolates with sequence lengths <1.4 Mb (*n*=6) and coverage <20× (*n*=15) were added to the dataset to examine potential variations in allele calling (core genome and whole genome) due to low values of these metrics. All plots used to show the correlations of the quality metrics (average read quality, average *de novo* coverage, sequence length, number of contigs, number of ambiguous base calls and N50 values) for all sequences and the percentage of core genome loci with allele calls (percentage core called) or number of wgMLST alleles with allele calls (present alleles) were created in R Studio v4.1.1 using the GGplot2 package [41].

### Sequence typing and cluster analysis using cgMLST, wgMLST and hqSNP

**Fig. 2.**
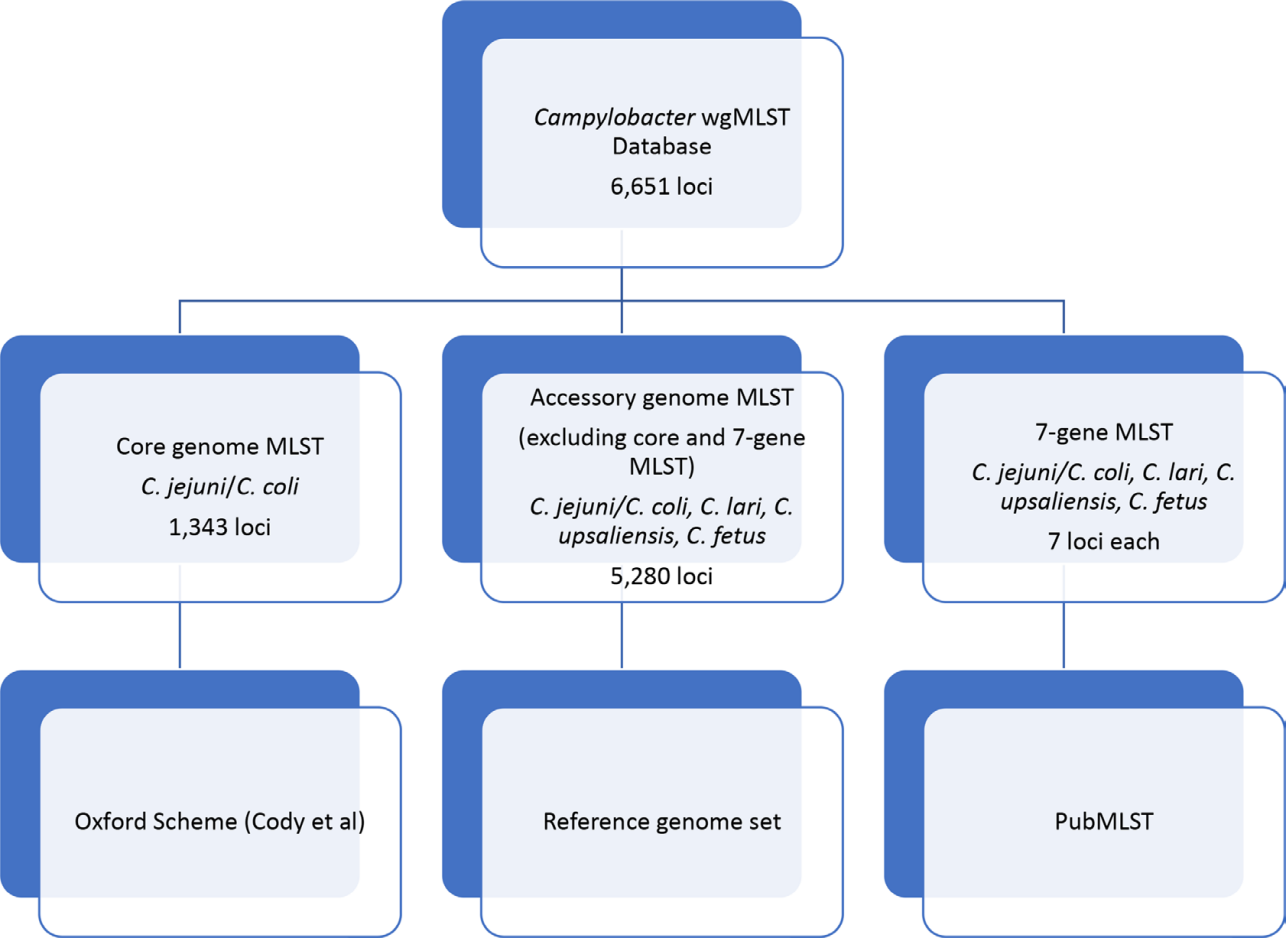
PulseNet *Campylobacter* schema development. Numbers of loci included within schemes are shown for the overall scheme, core genome, accessory genome (excluding core) and 7-gene MLST.

*In silico* 7-gene MLST analysis of sequences was performed using a feature in BioNumerics v7.6.3 which provides the 7-gene MLST STs from the *Campylobacter* MLST database (http://pubmlst.org/campylobacter). Cluster analysis in the *Campylobacter* National Database using cgMLST and wgMLST allele calls from loci identified in all 315 outbreak and sporadic isolate sequences (similarity coefficient of categorical values) was used to generate unweighted pair group method using average linkages (UPGMA) dendrograms. Loci without allele calls were ignored in pairwise sequence comparisons used to generate the dendrograms. The cgMLST and wgMLST UPGMA dendrograms were circularized and annotated using iTOL v6.4.2 [42]. The cgMLST scheme used in the BioNumerics database is available at http://pubmlst.org/campylobacter, and contains 1 343 *C *. *jejuni */* C. coli* loci [25]. The wgMLST scheme used in the BioNumerics database contained an additional 5 280 accessory loci found in *C. jejuni *, *C. coli *, *C. lari *, *C. fetus* and *C. upsaliensis* reference sequences as well as 7-gene MLST loci for *C. jejuni */* C. coli *, *C. lari *, *C. fetus* and *C. upsaliensis* from PubMLST (Fig. 2).

The hqSNP analysis was performed using Lyve-SET v1.1.4f (http://github.com/lskatz/Lyve-SET) to create maximum-likelihood trees. Specifically, SNPs were called using VarScan and had a minimum read consensus of 95 %, minimum read coverage of 20× and minimum flanking distance of 100 bp. Closed or outbreak-specific draft assemblies (assembled using SPAdes v.3.14.0) listed in Table 2 were used as reference sequences with plasmids masked using PlasFlow v1.1 and phages masked using the Lyve-SET workflow.

**Table 2.** Reference sequences used for hqSNP analysis

Outbreak	Strain	Closed or draft	Length (bp)	N50 (bp)	Contigs	BioSample	Assembly/SRR
0810PADBR-1	D7323	Draft	1 602 561	182 837	13	SAMN04545068	SRR3215124
1008MTDBR-1	2010D-8350	Draft	1 593 052	283 129	9	SAMN05226788	SRR3658031
1302AKDBB-1	2013D-9583	Draft	1 645 193	214 413	11	SAMN02650932	SRR1178523
1506UTDBR-1	D0133	Closed	1 766 499	1 766 499	1	SAMN16387699	CP063085
1509VTDBR-1	2015D-0147	Draft	1 631 767	151 547	20	SAMN04147047	SRR2561787
1510WIDBR-1	PNUSAC000138	Draft	1 602 515	295 596	10	SAMN04244354	SRR2916004
1602VTDBR-1	2016D-0045	Draft	1 629 630	160 975	15	SAMN04579139	SRR3291011
1607CADBR-1	D5663	Closed	1 630 187	1 630 187	1	SAMN03569365	CP068471
1609CODBR-1	2015D-0022	Closed	1 768 046	1 768 046	1	SAMN31367385	CP109649
1612OHDBR-1	B16-25748	Draft	1 654 468	214 000	10	SAMN06068091	SRR5076583
1708FLDBR-1a	PNUSAC002743	Draft	1 649 080	291 852	10	SAMN07646583	SRR6048555
1708FLDBR-1b	2016AY-1256	Draft	1 659 292	205 094	13	SAMN08098204	SRR6354026
1906NVDBR-1	PNUSAC015167	Draft	1 647 657	212 790	12	SAMN13897151	SRR10919697
1907VTDBR-1	PNUSAC010073	Draft	1 646 564	296 819	9	SAMN16428353	SRR12817698
2102NHDBR-1	PNUSAC020473	Draft	1 605 239	295 455	9	SAMN18046979	SRR13776832

Assembly accession numbers are provided for closed reference sequences generated on PacBio and uploaded to NCBI.

SRR identification numbers are used for draft Illumina sequences uploaded to NCBI.

bp, base pairs.

### Comparison of phylogenetic trees

To compare phylogenies generated from cgMLST, wgMLST and hqSNP data, Baker’s Gamma Indices [43] and pairwise cophenetic correlations coefficients [44] were generated from the BioNumerics v7.6.3 or LYVE-Set v1.1.4f Newick files using the dendextend package in R v4.1.2 (https://github.com/rstudio/rstudio). For both analyses, a value close to 1.0 is indicative of similar tree topology, which can be interpreted as strong concordance between workflows [43, 44].

### Genetic pairwise distance comparisons and linear regressions

Pairwise cgMLST and wgMLST allelic differences (*y*-axis) were plotted against their respective pairwise hqSNP differences (*x*-axis) for all outbreak-related isolates (entire clonal outbreaks or within clades designated in Table 1 for polyclonal outbreaks) and linear regression was performed. We determined the linear regression of each analysis to create trend lines and its formula (*y*=*mx*+*b*) with a slope (*m*) indicative of differences in pairwise distances between analysis methods, *y*-intercept (*b*) to determine the pairwise cg/wgMLST allele differences when hqSNP differences were 0 or pairwise wgMLST allele differences when cgMLST allele differences were 0, and an *R* ^2^ value as a statistical indication of fit to the linear regression trend line. Pearson correlation coefficients were calculated to compare genetic distance correlations between cgMLST, wgMLST and hqSNP analysis methods.

### Accession numbers

Sequence reads for all 315 outbreak and sporadic isolate sequences were deposited in the Sequence Read Archive at the National Center for Biotechnology Information with the accession numbers shown in Table S1.

## Results

### Sequence quality assessment

**Fig. 3.**
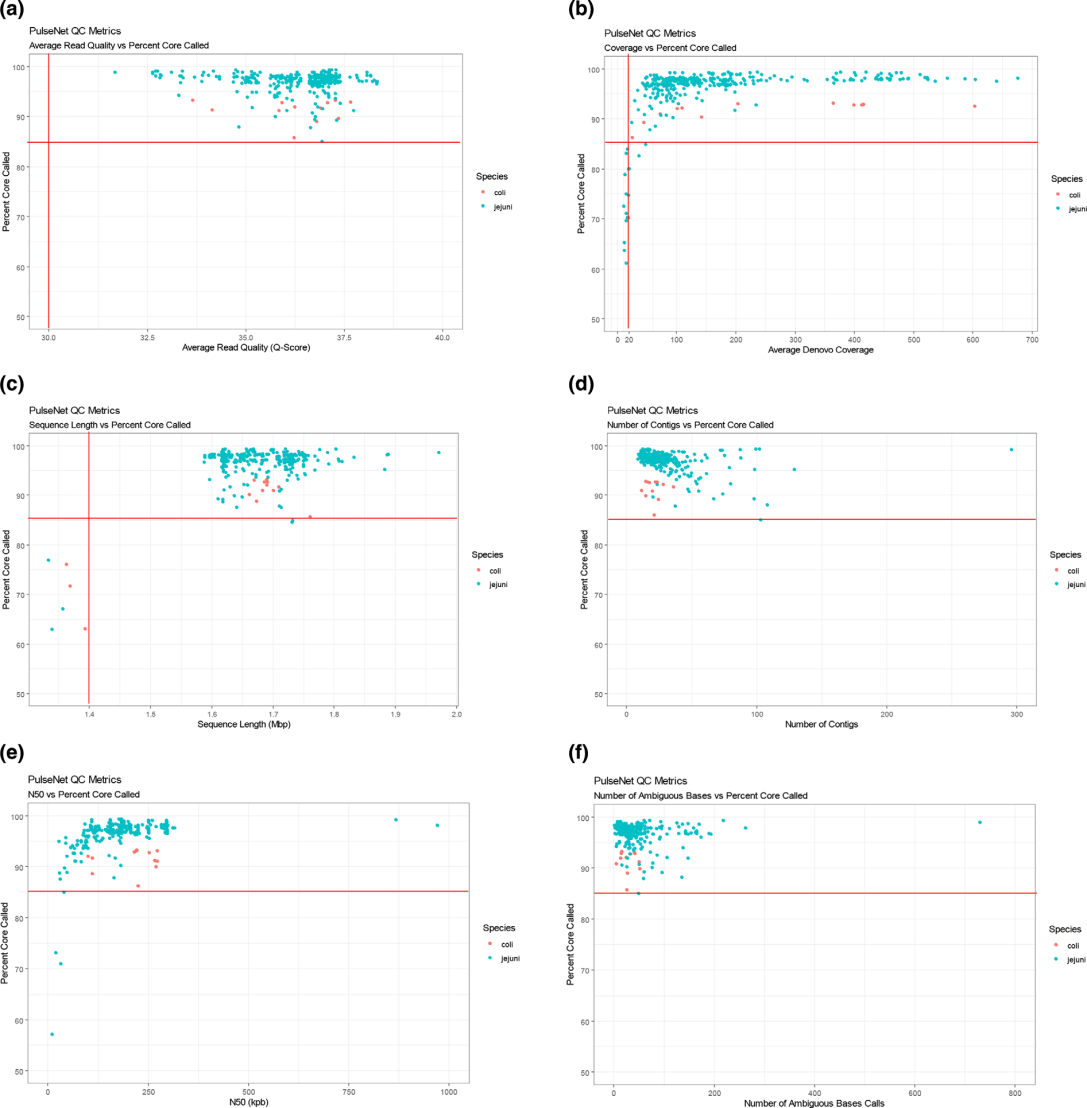

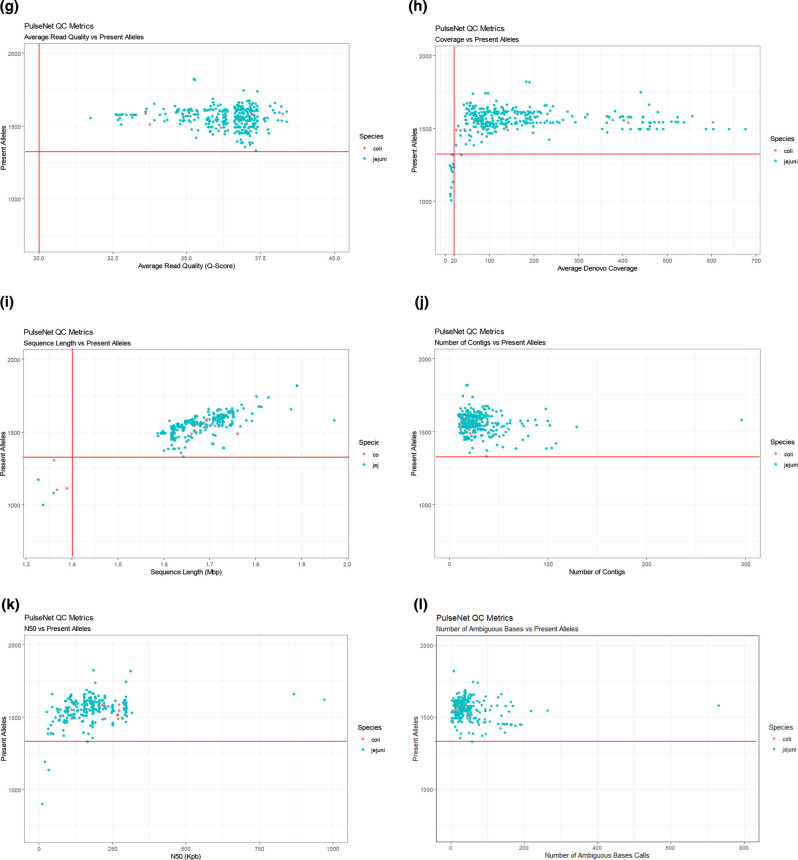
Sequence quality metrics and corresponding cgMLST allele calls (percent core called, **a–f**) or wgMLST allele calls (present alleles, **g–l**) for all 336 sequenced *Campylobacter* isolates. An additional 15 sporadic isolates with sequence length <1.4 Mb and an additional six sporadic isolates with an average *de novo* coverage <20× were added to the respective datasets. *C. jejuni* and *C. coli* isolate sequences are coloured according to the key. PulseNet quality metric thresholds are indicated by the red lines.

The average read quality (all sequences had read quality Q-scores ≥32) did not affect the core genome and whole genome allele calling (Fig. 3a, g). Likewise, the number of contigs and number of ambiguous base calls did not affect the core genome and whole genome allele calling (Fig. 3). However, low average *de novo* coverage (<20×), low sequence length (<1.4 Mb) and low N50 values (<20 000) resulted in low core genome (<85 % core present called) and whole genome (<1 300 present alleles) allele calls (Fig. 3b, c, e, h, i, k). These results show that setting the sequence length and coverage thresholds to >1.4 Mb and >20×, respectively, is necessary to meet cgMLST allele calling thresholds. There were no sequences available with sequence length >2.2 Mb to examine the effects of high sequence length on allele calling as sequence lengths >2.2 Mb probably indicate contamination and such sequences would not be included for further analysis. Even though thresholds for number of contigs, number of ambiguous base calls and N50 values are not used in PulseNet for sequence quality determinations, values ≥150, ≥500 and ≤50 000 bp for these metrics, respectively, can indicate sequence quality issues in some cases and are considered conditional cutoff values (Fig. 1).

### Epidemiological concordance of WGS data analysis methods

**Fig. 4.**
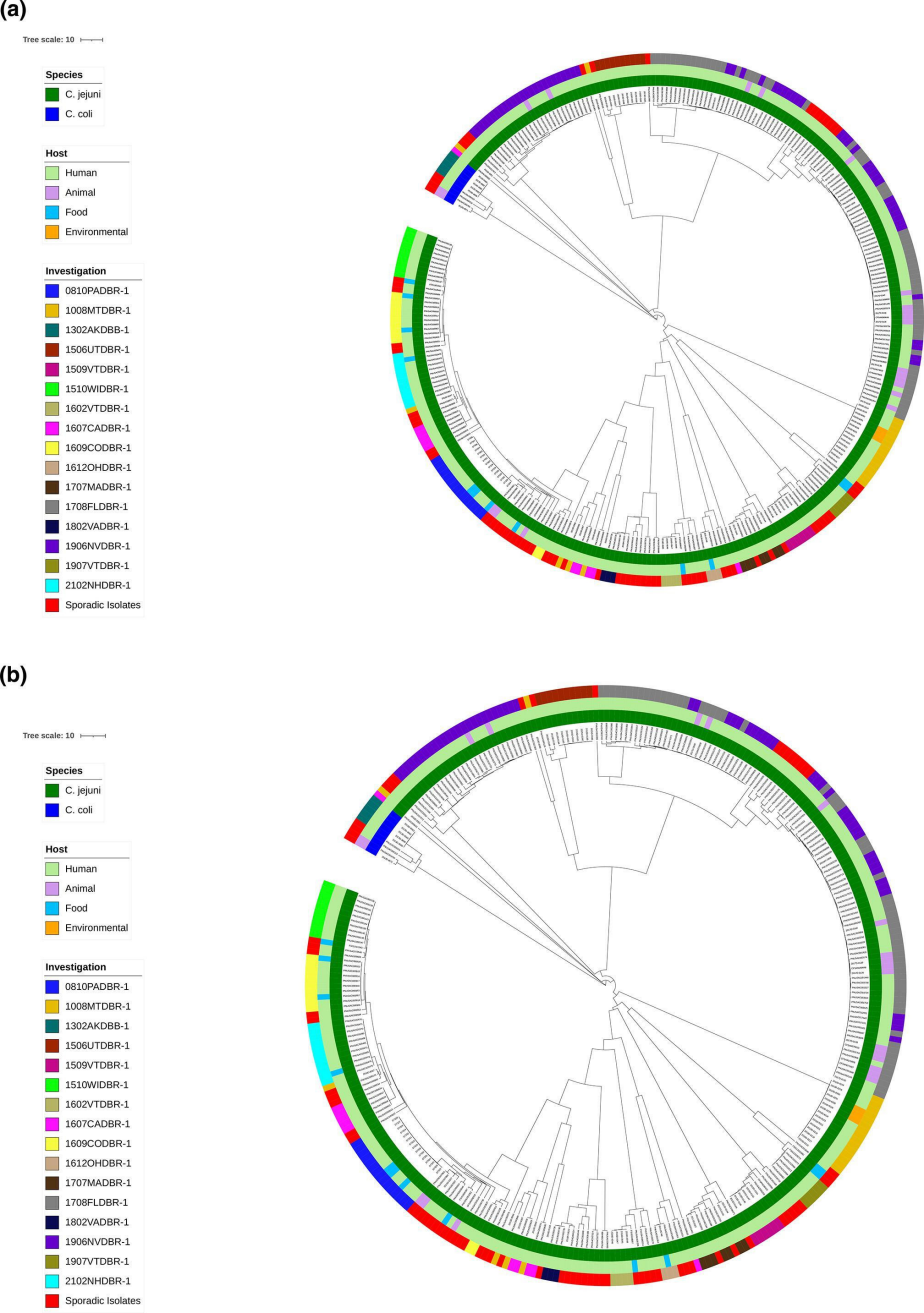
UPGMA phylogenic dendrograms with outbreak (237 C. *jejuni* and five *C. coli *) and sporadic (69 C. *jejuni* and four *C. coli *) sequences based on cgMLST (**a**) and wgMLST (**b**) schemes in BioNumerics v7.6.3. Dendrograms were circularized and annotated using IToL. Inner circle represents species of isolates, second ring represents source for each isolate, and the last ring represents the outbreak investigation each isolate belongs to or the sporadic isolates. Colour codes for species, source, and outbreak or sporadic isolates are shown in the key.

The clustering of *Campylobacter* isolates by WGS (hqSNP, cgMLST and wgMLST analysis) was concordant with epidemiological information of the corresponding cases for all outbreaks in this study (Table 1). Within nine clonal outbreaks investigated in this study, few genetic distances (≤5 SNPs, ≤10 cgMLST alleles, and ≤12 wgMLST alleles) among the outbreak isolates were observed. The other nine outbreaks contained multiple strains of *C. jejuni* and/or *C. coli* and were included in the same outbreaks based on epidemiological information collected during the investigations; multiple cgMLST clades are indicated next to the outbreak code (i.e., a, b, c) in Table 1. The sources of these outbreaks were unspecified water, irrigation water, Mexican taqueria, raw milk, chicken liver and pet store puppies (Table 1). There were two to three WGS clades (not including singletons) per polyclonal outbreak with varying within-clade genetic distances (≤124 SNPs, ≤59 cgMLST alleles and ≤78 wgMLST alleles), including the puppy outbreaks 1708FLDBR-1 and 1906NVDBR-1. Removing the zoonotic puppy outbreaks, within-clade genetic distances were ≤27 SNPs, ≤12 cgMLST alleles and ≤17 wgMLST alleles. Most (68/73) of the sporadic isolates were genetically distinct from the outbreak isolates by both cgMLST and wgMLST analysis (Fig. 4a, b). However, 5/73 sporadic isolates were closely related (≤11 cgMLST alleles) to outbreak isolates.

### Comparison of WGS phylogenetic analyses

The minimum and maximum cgMLST and wgMLST allelic and hqSNP distances of the within-clade outbreak and corresponding sporadic isolate sequences are shown in Table 1. The maximum within-clade cgMLST and wgMLST allele differences were identical in 11/25 clonal outbreaks or polyclonal outbreak clades while there were larger wgMLST allele differences compared to cgMLST allele differences in 14/25 outbreak clades. Including the outbreak clade-specific sporadic isolate sequences, the maximum number of wgMLST allele differences was always greater than the cgMLST allele differences. In 10/25 outbreak clades, the maximum within-clade SNP differences were higher than wgMLST allele differences; however, isolates in 15/25 outbreak clades displayed the same or fewer SNP differences compared to the wgMLST allele differences. After including the sporadic isolate sequences, the maximum number of hqSNP differences was greater than the wgMLST allele differences in all except one instance. The maximum SNP differences were the same or less than the cgMLST allele differences for 13/25 outbreak clades, which was not the case after inclusion of the sporadic isolates.

**Fig. 5.**
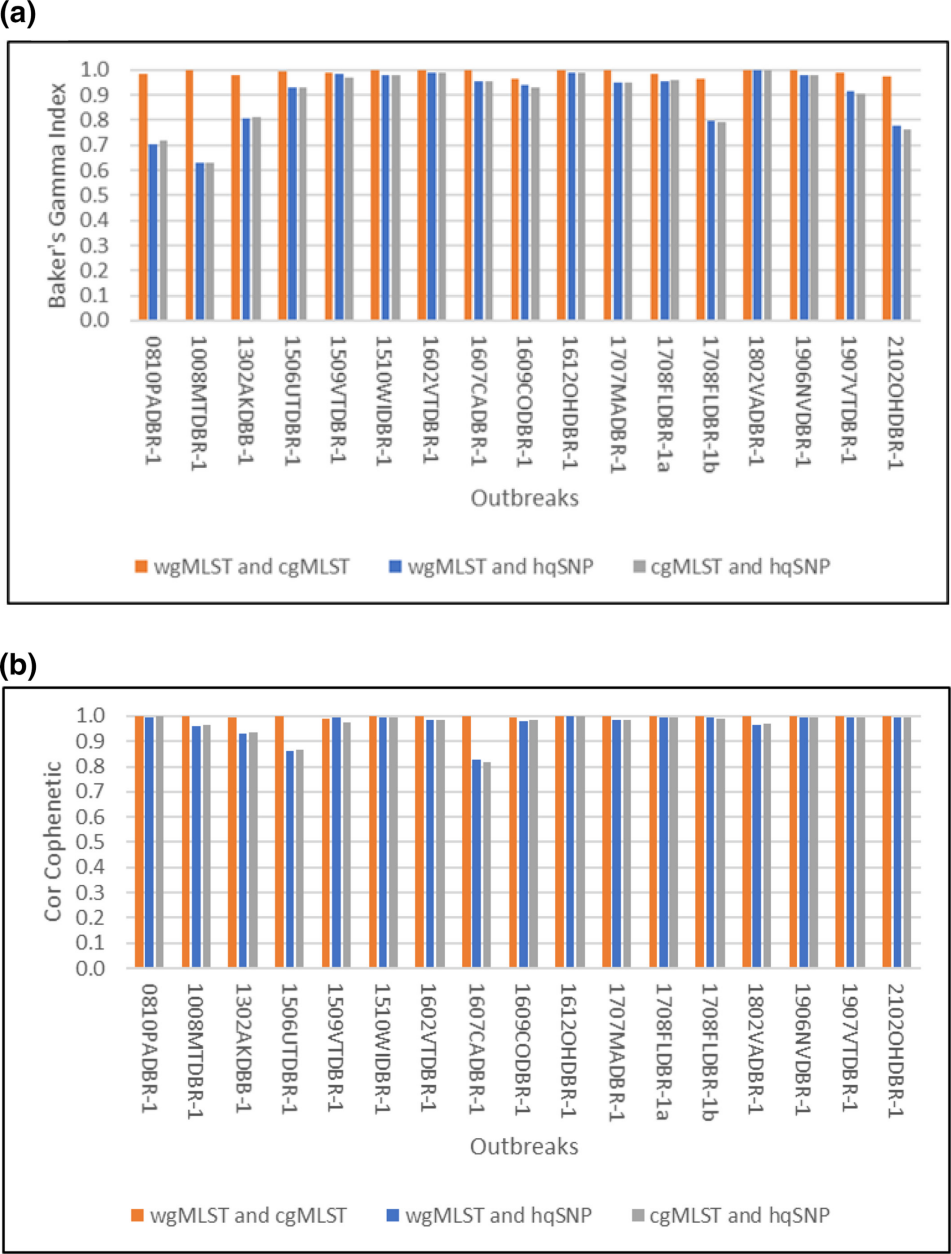
Bar graphs of Baker’s gamma index (**a**) and cophenetic correlation coefficient (**b**) for all outbreak+sporadic isolate datasets in this study. Comparisons of wgMLST and cgMLST dendrograms are in orange, comparisons of wgMLST and hqSNP dendrograms are in blue, and comparisons of cgMLST and hqSNP dendrograms are in silver. Values close to 1 indicate that the two analysis methods compared are more similar.

Baker’s gamma index (BGI) and cophenetic correlation coefficient were calculated to quantify the similarities between the cgMLST, wgMLST and hqSNP dendrograms for the outbreak and sporadic isolate datasets. Outbreak 1708FLDBR-1 was split into two hqSNP analyses (1708FLDBR-1a and 1708FLDBR-1b) resulting in 17 outbreak and sporadic isolate datasets. The median BGI was highest when comparing cgMLST and wgMLST for all 17 datasets (Table S2, Fig. 5a) (0.996, range: 0.965–1.000), which is concordant with the dendrograms illustrated in Fig. 4(a, b) and showing that cgMLST and wgMLST analyses clustered the isolates similarly. For 12 out of the 17 datasets, the median BGI was high comparing cgMLST vs. hqSNP (0.966, range: 0.904–0.999) and wgMLST vs. hqSNP (0.966, range: 0.914–1.000). The median BGI was lower for the other five datasets comparing cgMLST vs. hqSNP (0.764, range: 0.630–0.813) and wgMLST vs. hqSNP (0.775, range: 0.630–0.808). The median cophenetic correlation coefficient was highest comparing cgMLST and wgMLST for all datasets (Table S2, Fig. 5b) (1.000, range: 0.991–1.000) followed by wgMLST vs. hqSNP (0.992, range: 0.828–1.000) and cgMLST vs. hqSNP (0.986, range: 0.818–0.998). CgMLST and hqSNP dendrograms were less similar for polyclonal 1506UTDBR-1 and 1607CADBR-1 outbreaks and sporadic isolate datasets (cophenetic correlation coefficients: 0.812–0.864) compared to the remaining outbreak datasets (cophenetic correlation coefficients: >0.933). Similar cophenetic correlation coefficients were observed comparing wgMLST to hqSNP dendrograms for these two datasets (0.828–0.860) compared to the remaining datasets (>0.931).

### Genetic pairwise distance comparisons and linear regressions

**Fig. 6.**
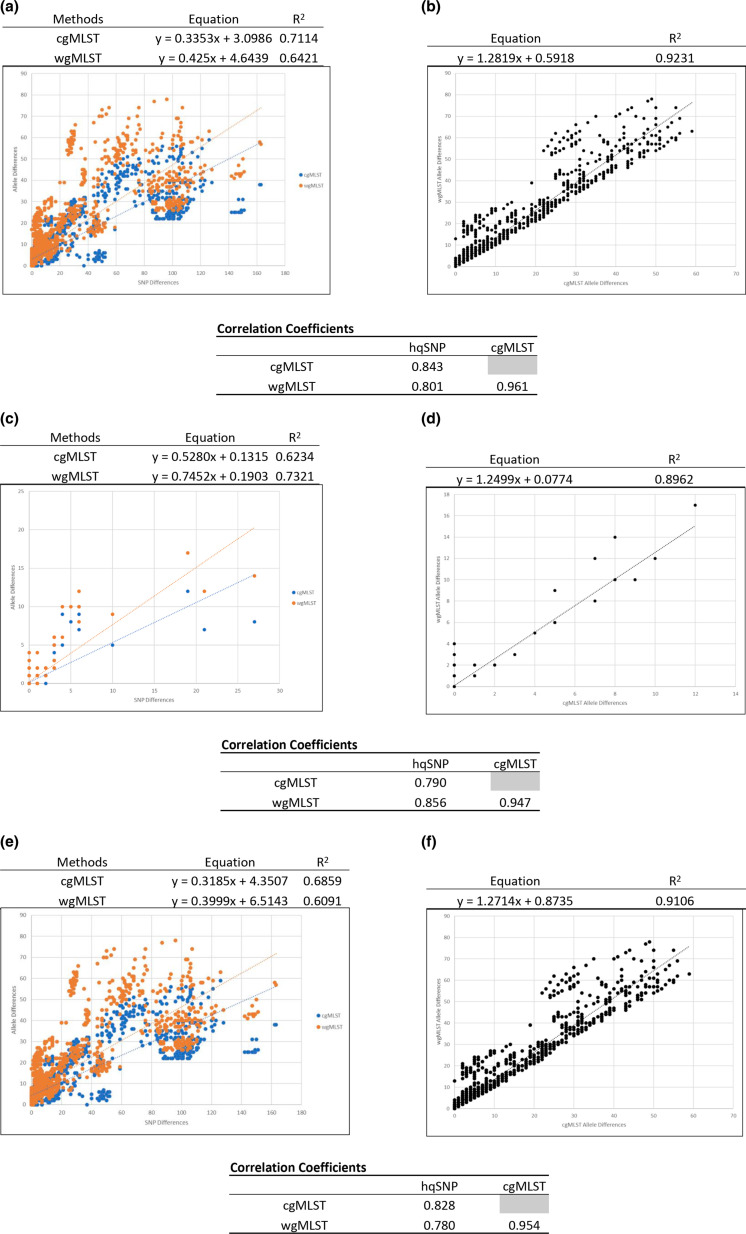
Scatterplot of all outbreak-related pairwise differences of cgMLST and wgMLST against hqSNP (**a, c, e**) and cgMLST against wgMLST (**b, d, f**). Scatterplots for all within-clade outbreak isolates are shown in (a) and (b), excluding puppy outbreak isolates (1708FLDBR-1 and 1906NVDBR-1) are shown in (c) and (d), and only puppy outbreak isolates are shown in (e) and (f). A simple linear regression analysis was performed for each comparison to produce a best-fit line; the formulas (*y*=*mx*+*b*) and variabilities (***r* ^2^**) are shown on the figure. Pearson correlation coefficients were also calculated and are displayed on the figure.

All outbreak-related pairwise allelic genetic differences (entire clonal outbreaks or within-clades designated in Table 1 for polyclonal outbreaks) generated by cgMLST and wgMLST were combined and plotted against their respective hqSNP differences in the scatterplot illustrated in Fig. 6(a). The *R* ^2^ values comparing cgMLST and wgMLST allelic differences to the hqSNP pairwise SNP differences were 0.7114 and 0.6421, respectively. The slopes of the linear regressions were as follows: cgMLST (0.3353) < wgMLST (0.425), indicating that there are slightly higher allele differences using wgMLST compared to hqSNP than cgMLST to hqSNP. Note that the slopes are <1, demonstrating that there are more pairwise SNP differences in the dataset compared to cg/wgMLST allelic differences. The *y*-intercepts comparing cgMLST and wgMLST allelic differences to the hqSNP pairwise SNP differences were 3.0986 and 4.6439, respectively, illustrating that on average sequences that were zero SNPs different were approximately three and five alleles different. The Pearson correlation coefficients were 0.843 for cgMLST and 0.801 for wgMLST compared to hqSNP analysis. The pairwise within-clade outbreak allelic genetic differences generated by cgMLST were also plotted against their respective wgMLST differences in the scatterplot illustrated in Fig. 6(b). There was a high correlation between cgMLST and wgMLST (*R* ^2^=0.9231) allelic differences for these sequences. The slope of the linear regression was 1.2819, indicating that there were more pairwise wgMLST allele differences than cgMLST allele differences in the dataset. The *y*-intercept comparing cgMLST and wgMLST allelic differences was 0.5918, illustrating that on average sequences that were zero core genome alleles different were approximately zero whole genome alleles different, which is expected comparing cgMLST and wgMLST methods. The Pearson correlation coefficient was 0.961.

The large zoonotic outbreak datasets (1708FLDBR-1 and 1906NVDBR-1) were removed from the analysis and the linear regression models recalculated to determine if linear regression values were different when calculated primarily on foodborne outbreaks (Fig. 6c, d). In the scatterplots excluding the zoonotic outbreak isolates, the correlations between cgMLST and wgMLST allelic differences compared to the hqSNP pairwise differences were lower than the first dataset (*R* ^2^=0.6234 and 0.7321) (Fig. 6c). The slopes of the linear regressions were as follows: cgMLST (0.5280) and wgMLST (0.7452). The *y*-intercepts comparing cgMLST and wgMLST allelic differences to the hqSNP pairwise differences were 0.1315 and 0.1903, illustrating that on average, sequences that were zero SNPs different were approximately zero alleles different, which is expected comparing two different WGS analysis methods on the same dataset. The Pearson correlation coefficients were 0.790 for cgMLST and 0.856 for wgMLST compared to hqSNP analysis. There was a high correlation between cgMLST and wgMLST (*R* ^2^=0.8962) allelic differences for these sequences (Fig. 6d). The slope of the linear regression was 1.2499. The *y*-intercept comparing cgMLST and wgMLST was 0.0774, illustrating that sequences that were zero core genome alleles different were approximately zero whole genome alleles different. The Pearson correlation coefficient was 0.947.

The linear regression models including only the large zoonotic outbreak datasets (1708FLDBR-1 and 1906NVDBR-1) were analysed to determine if linear regression values were different when calculated primarily on these outbreaks (Fig. 6e, f). The correlations between cgMLST and wgMLST allelic differences compared to the hqSNP pairwise differences were lower than the first and second datasets (*R* ^2^=0.6859 and 0.6091) (Fig. 6e). The slopes of the linear regressions were as follows: cgMLST (0.3185) < wgMLST (0.3999). The *y*-intercepts comparing cgMLST and wgMLST allelic differences to the hqSNP pairwise differences were 4.3507 and 6.5143 illustrating that, on average, sequences that were zero SNPs different were approximately four and seven alleles different comparing two different WGS analysis methods on the same dataset. The Pearson correlation coefficients were 0.828 for cgMLST and 0.780 for wgMLST compared to hqSNP analysis. There was a high correlation between cgMLST and wgMLST (*R* ^2^=0.9106) allelic differences for these sequences (Fig. 6f). The slope of the linear regression was 1.2714. The *y*-intercept comparing cgMLST and wgMLST was 0.8735, illustrating that on average sequences that were zero core genome alleles different were approximately one whole genome allele different. The Pearson correlation coefficient was 0.954. Overall, there was a higher concordance between methods with the zoonotic outbreak isolates included in the analysis.

## Discussion

In this study, we showed that cgMLST analysis of WGS data was highly concordant with epidemiological information linking cases in *Campylobacter* outbreaks and mostly differentiated sporadic isolates that were indistinguishable by PFGE or 7-gene MLST to outbreak isolates. There were a few sporadic isolates that were within 10 cgMLST alleles to outbreak isolates. A United States Department of Agriculture (USDA) beef isolate was 7–9 cgMLST alleles different from the 1510WIDBR-1 outbreak isolates; however, this beef isolate was from Arizona and obtained 5–6 months before the local Wisconsin church fundraiser outbreak was identified. This isolate was also 11–12 wgMLST alleles and 20–22 SNPs from the outbreak isolates, which is beyond the 10-allele or SNP threshold for including isolates in an outbreak cluster. Human isolates from New York and Maryland were 6–11 cgMLST alleles different from the 1802VTDBR-1 outbreak isolates. These sporadic isolates were recovered only a month after the 1802VTDBR-1 outbreak isolates, but wgMLST and hqSNP analysis further differentiated these isolates outside of the outbreak threshold (20–25 wgMLST alleles and 12–15 SNPs). A Maryland human isolate was 5–7 cgMLST alleles from the 1509VTDBR-1 outbreak isolates. This isolate was obtained two months after the local Vermont poultry outbreak was identified; however, it was further differentiated by wgMLST (31–35 alleles) and hqSNP (76–96 SNPs) analyses from the outbreak isolates. These instances demonstrate that wgMLST and hqSNP analyses can further differentiate isolates that cluster by cgMLST (≤10 alleles different) during outbreak investigations and isolates linked together with epidemiological information clustered by cgMLST. Further, the sporadic isolates may have been temporally or geographically separated from the outbreak isolates, missed during the initial outbreak investigation, or excluded from the outbreak due to lack of epidemiological information linking the isolates together. Interestingly, a USDA chicken isolate was only 4–5 cgMLST alleles, 5–6 wgMLST alleles and 7–8 SNPs from the 1907VTDBR-1 outbreak isolates, which is the only instance in this study that further wgMLST and hqSNP analyses would not separate a sporadic isolate beyond 10 alleles or SNPs from the outbreak isolates. This chicken isolate was from Virginia and obtained around 1 year after the local Vermont poultry outbreak was identified, but it is possible that these two sources contained the same circulating *C. jejuni* strain.

The allelic threshold used to determine whether sporadic isolates were closely genetically related to outbreak isolates during foodborne outbreaks (i.e. ≤10 cgMLST alleles different) was narrower than the maximum allelic differences observed during the zoonotic *Campylobacter* outbreaks linked to pet store puppies. Allelic thresholds are used to initially link potential outbreak isolates, and epidemiological and additional genetic information, such as antibiotic resistance genes, can be used to further define potential outbreak isolates [14].

The three WGS analysis workflows had high concordance in clustering the *Campylobacter* isolates in epidemiologically linked outbreaks, regardless of whether they are SNP-based (hqSNP workflow) or MLST-based (cg/wgMLST workflows). A larger number of outbreak isolates displayed high SNP-based vs. MLST-based concordance using the cophenetic correlation coefficient (100 % of outbreaks with values >0.8) than the BGI (77 % of outbreaks with values >0.8). BGI measures the similarities between two dendrograms [43] and the cophenetic correlation coefficient measures the similarities in pairwise distances of dendrograms between the data points [44]; therefore, these metrics calculate the similarities between the WGS analysis methods differently but both statistical methods show that hqSNP, cgMLST and wgMLST analyses created similar tree topologies. Minor differences in cophenetic correlation coefficients between cg/wgMLST and hqSNP phylogenetic trees may reflect the differences in clustering techniques used, as MLST-based dendrograms were created using UPGMA while SNP-based dendrograms were created using the maximum likelihood method. Even though there was high concordance in clustering of outbreak isolates using SNP-based and MLST-based methods, there was some discrepancy in the pairwise differences between outbreak isolates using these WGS analysis methods, as indicated by the lower *R* ^2^ and Pearson correlation coefficient values. Pairwise allele differences were higher than SNP differences for isolates in 9/25 outbreak clades. This is not surprising since new alleles are called due to SNPs, insertions or deletions in coding regions whereas hqSNP analysis only counts SNPs against a reference genome with phages and plasmids masked [36]. Recombination and horizontal gene transfer via plasmids and phages play a major role in *C. jejuni* and *C. coli* evolution, particularly when multiple strains of *Campylobacter* are located within the same animal host [45–51]. Pairwise SNP differences were also much higher than allele differences for isolates in 9/25 outbreak clades. This may be due to several reasons, including SNPs in the intergenic region, multiple SNPs within a single gene (a single allelic difference may contain multiple SNP differences) and SNPs detected in genomic regions outside the cg/wgMLST schemes.

There was a high correlation between cgMLST and wgMLST analyses of the isolates as shown by the circularized dendrograms, BGI values, cophenetic correlation coefficients, and scatterplots (high *R* ^2^ values and Pearson correlation coefficients). In addition, the *y*-intercept value was <1, showing that sequences that had zero pairwise cgMLST allele differences also had zero pairwise wgMLST allele differences, as expected. These results are similar to those of a previous study by Nenning *et al*. that showed cgMLST and wgMLST analysis methods clustered *Campylobacter* strains similarly [52]. The slope of the linear regression model was approximately 1.25, indicating that there were about 1.25 pairwise wgMLST allelic differences for every cgMLST allele difference observed. In 14 outbreaks or outbreak clades, the pairwise differences were higher for wgMLST compared to cgMLST. Most probably, these pairwise differences were in the additional 5 280 accessory loci that are part of the wgMLST scheme.

This study demonstrated some of the limitations of the cgMLST scheme developed by Cody *et al*. [25], since only 88 % of our isolates had allele calls in 95 % or greater cgMLST loci. This limitation may have been caused by the fact that the isolates used to build and validate the scheme by Cody *et al*. were from the UK while in our study the isolates were from the USA. There is likely to be more variation in the presence of core genome loci in *Campylobacter* strains in the USA. The wgMLST scheme used in this study was developed using curated reference genomes from a variety of sources and has been first published here.

In conclusion, we demonstrated that we were able to cluster *C. jejuni* and *C. coli* isolates in concordance with epidemiological data using WGS-based analysis methods compared to traditional subtyping methods. Discrepancies between allele- and SNP-based approaches may reflect the differences between how genomic variation (SNPs and indels) is captured between the two methods. We found that cgMLST is sufficient for *C. jejuni* and *C. coli* outbreak detection and analysis while wgMLST can be used to further differentiate isolates if needed.

## Supplementary Data

Supplementary material 1

Supplementary material 2
