# Supplementary material for: Evaluation of core genome and whole genome multilocus sequence typing schemes for Campylobacter jejuni and Campylobacter coli outbreak detection in the USA
Source: Microb Genom. 2023 May 3;9(5):mgen001012. doi: 10.1099/mgen.0.001012 (PMC10272873; doi:10.1099/mgen.0.001012)
Supplement: Supplementary material 1 [file mgen-9-1012-s001.pdf]

Supplemental Table 1. Isolates used in the study.

Outbreak	Strain	Source State	Species	Year of Isolation	Source	MLST ST	PFGE-SmaI	PFGE-KpnI	Biosample No.	SRR No.
0810PADBR-1	D7316	PA	jejuni	2008	Raw Milk	ST8536	DBRS16.0008	DBRK02.0028	SAMN04545269	SRR3216133
0810PADBR-1	D7319	PA	jejuni	2008	Stool	ST8536	DBRS16.0008	DBRK02.0028	SAMN04545132	SRR3215210
0810PADBR-1	D7320	PA	jejuni	2008	Stool	ST8536	DBRS16.0008	DBRK02.0028	SAMN04545292	SRR3216186
0810PADBR-1	D7321	PA	jejuni	2008	Stool	ST8536	DBRS16.0008	DBRK02.0028	SAMN04545069	SRR3215135
0810PADBR-1	D7322	PA	jejuni	2008	Stool	ST8536	DBRS16.0008	DBRK02.0028	SAMN04545131	SRR3215209
0810PADBR-1	D7323	PA	jejuni	2008	Stool	ST8536	DBRS16.0008	DBRK02.0028	SAMN04545068	SRR3215124
0810PADBR-1	D7324	PA	jejuni	2008	Stool	ST8536	DBRS16.0008	DBRK02.0028	SAMN04545067	SRR3215123
0810PADBR-1	D7326	PA	jejuni	2008	Stool	ST8536	DBRS16.0008	DBRK02.0028	SAMN05226810	SRR3658064
0810PADBR-1	D7327	PA	jejuni	2008	Stool	ST8536	DBRS16.0008	DBRK02.0028	SAMN04545133	SRR3215211
0810PADBR-1	D7328	PA	jejuni	2008	Stool	ST8536	DBRS16.0008	DBRK02.0028	SAMN04545064	SRR3215108
0810PADBR-1	D7329	PA	jejuni	2008	Stool	ST8536	DBRS16.0008	DBRK02.0028	SAMN04545066	SRR3215107
0810PADBR-1	D7331	PA	jejuni	2008	Stool	ST8536	DBRS16.0008	DBRK02.0028	SAMN04545062	SRR3214715
0810PADBR-1	D7332	PA	jejuni	2008	Stool	ST8536	DBRS16.0008	DBRK02.0028	SAMN05226811	SRR3658065
0810PADBR-1	D7333	PA	jejuni	2008	Raw Milk	ST8536	DBRS16.0008	DBRK02.0028	SAMN04545255	SRR3216118
0810PADBR-1	D7334	PA	jejuni	2008	Raw Milk	ST8536	DBRS16.0008	DBRK02.0028	SAMN04545316	SRR3216366
Non-0810PADBR01	2014D-0207	VT	jejuni	2010	Stool	ST3599	DBRS16.0008	DBRK02.0028	SAMN04545030	SRR3214556
Non-0810PADBR01	PNUSAC007697	WY	jejuni	2010	Stool	ST8	DBRS16.0008	DBRK02.0028	SAMN10721955	SRR8428233
Non-0810PADBR01	PNUSAC007883	WY	jejuni	2009	Ovine Liver	ST8	DBRS16.0008	DBRK02.0028	SAMN10852014	SRR8511717
Non-0810PADBR01	PNUSAC007884	WY	jejuni	2009	Ovine Lung	ST8	DBRS16.0008	DBRK02.0028	SAMN10852013	SRR8511718
1008MTDBR-1	2010D-8214	MT	jejuni	2010	Stool	ST6839	DBRS16.0054	DBRK02.0665	SAMN05226777	SRR3657947
1008MTDBR-1	2010D-8215	MT	jejuni	2010	Stool	ST6839	DBRS16.0054	DBRK02.0665	SAMN05226778	SRR3657948
1008MTDBR-1	2010D-8216	MT	jejuni	2010	Stool	ST6839	DBRS16.0054	DBRK02.0665	SAMN05226779	SRR3657949
1008MTDBR-1	2010D-8217	MT	jejuni	2010	Stool	ST6839	DBRS16.0054	DBRK02.0665	SAMN05226781	SRR3657951
1008MTDBR-1	2010D-8218	MT	jejuni	2010	Stool	ST6839	DBRS16.0054	DBRK02.0665	SAMN05226780	SRR3657950
1008MTDBR-1	2010D-8219	MT	jejuni	2010	Stool	ST6839	DBRS16.0054	DBRK02.0665	SAMN05226782	SRR3657952
1008MTDBR-1	2010D-8221	MT	jejuni	2010	Stool	ST6839	DBRS16.0054	DBRK02.0665	SAMN05226783	SRR3657959
1008MTDBR-1	2010D-8222	MT	jejuni	2010	Stool	ST6839	DBRS16.0054	DBRK02.0665	SAMN05226784	SRR3658017
1008MTDBR-1	2010D-8223	MT	jejuni	2010	Stool	ST6839	DBRS16.0054	DBRK02.0665	SAMN05226785	SRR3658018
1008MTDBR-1	2010D-8224	MT	jejuni	2010	Stool	ST6839	DBRS16.0054	DBRK02.0665	SAMN05226786	SRR3658019
1008MTDBR-1	2010D-8225	MT	jejuni	2010	Stool	ST6839	DBRS16.0054	DBRK02.0665	SAMN05226787	SRR3658020
1008MTDBR-1	2010D-8226	MT	jejuni	2010	Water	ST6839	DBRS16.0054	DBRK02.0665	SAMN05226822	SRR3658076
1008MTDBR-1	2010D-8290	MT	jejuni	2010	Septic Cesspool	ST6839	DBRS16.0054	DBRK02.0665	SAMN05226823	SRR3658077
1008MTDBR-1	2010D-8291	MT	jejuni	2010	Septic Cesspool	ST6839	DBRS16.0054	DBRK02.0665	SAMN05226824	SRR3658078
1008MTDBR-1	2010D-8346	MT	jejuni	2010	Stool	ST21	DBRS16.0206	DBRK02.0135	SAMN05226775	SRR3657900
1008MTDBR-1	2010D-8347	MT	jejuni	2010	Stool	ST8	DBRS16.0008	DBRK02.0148	SAMN03565942	SRR1993248
1008MTDBR-1	2010D-8349	MT	jejuni	2010	Stool	ST982	DBRS16.0099	DBRK02.0021	SAMN05226776	SRR3657938
1008MTDBR-1	2010D-8350	MT	jejuni	2010	Stool	ST6839	DBRS16.0054	DBRK02.0665	SAMN05226788	SRR3658031
1008MTDBR-1	2010D-8351	MT	jejuni	2010	Stool	ST468	DBRS16.0093	DBRK02.0507	SAMN05226789	SRR3658043
1008MTDBR-1	2010D-8387	MT	jejuni	2010	Stool	ST11241	DBRS16.0240	DBRK02.0325	SAMN05226790	SRR3658044
1008MTDBR-1	2010D-8388	MT	jejuni	2010	Stool	ST45	DBRS16.0042	DBRK02.0176	SAMN05226791	SRR3658045
1302AKDBB-1	2013D-9562	AK	coli	2013	Stool	ST1068	DBBS16.0385	DBBK02.0165	SAMN02650930	SRR1178528
1302AKDBB-1	2013D-9563	AK	coli	2013	Stool	ST1068	DBBS16.0385	DBBK02.0165	SAMN02650928	SRR1178526
1302AKDBB-1	2013D-9582	AK	coli	2013	Stool	ST1068	DBBS16.0385	DBBK02.0165	SAMN02650931	SRR1178525
1302AKDBB-1	2013D-9583	AK	coli	2013	Stool	ST1068	DBBS16.0385	DBBK02.0165	SAMN02650932	SRR1178523
1302AKDBB-1	2013D-9606	AK	coli	2013	Stool	ST1068	DBBS16.0385	DBBK02.0165	SAMN02650929	SRR3214483
Non-1302AKDBB-1	2013D-9571	WY	coli	2013	Bovine Feces	ST1068	DBBS16.0385	DBBK02.0165	SAMN03566577	SRR1993564
Non-1302AKDBB-1	PNUSAC000219	MN	coli	2015	Stool	ST1068	DBBS16.0032	NA	SAMN04356531	SRR3057086
Non-1302AKDBB-1	PNUSAC000315	UT	coli	2016	Stool	ST1068	DBBS16.0032	NA	SAMN04849715	SRR4045540
Non-1302AKDBB-1	PNUSAC003700	WY	coli	2011	Bovine Feces	ST1068	DBBS16.0385	DBBK02.0165	SAMN08455613	SRR6667744
1506UTDBR-1	2015D-0159	UT	jejuni	2015	Stool	ST45	DBRS16.0035	DBRK02.0164	SAMN04545080	SRR3215151
1506UTDBR-1	2015D-0160	UT	jejuni	2015	Stool	ST11224	DBRS16.0035	DBRK02.0164	SAMN04545079	SRR3215150
1506UTDBR-1	2015D-0161	UT	jejuni	2015	Stool	ST11224	DBRS16.0035	DBRK02.0164	SAMN04545078	SRR3215148
1506UTDBR-1	2015D-0162	UT	jejuni	2015	Stool	ST11224	DBRS16.0035	DBRK02.0164	SAMN04545076	SRR3215147
1506UTDBR-1	2015D-0163	UT	jejuni	2015	Stool	ST11224	DBRS16.0035	DBRK02.0164	SAMN04545075	SRR3215146
1506UTDBR-1	2015D-0164	UT	jejuni	2015	Stool	ST11224	DBRS16.0035	DBRK02.0164	SAMN04545033	SRR3214563
1506UTDBR-1	2015D-0165	UT	jejuni	2015	Stool	ST11224	DBRS16.0035	DBRK02.0164	SAMN04545074	SRR3215145
1506UTDBR-1	2015D-0166	UT	jejuni	2015	Stool	ST11224	DBRS16.0035	DBRK02.0164	SAMN04545073	SRR3215143
1506UTDBR-1	2015D-0168	UT	jejuni	2015	Stool	ST11224	DBRS16.0035	DBRK02.0164	SAMN04545072	SRR3215142
1506UTDBR-1	2015D-0169	UT	jejuni	2015	Stool	ST45	DBRS16.0035	DBRK02.0164	SAMN04545071	SRR3215141
Non-1506UTDBR-1	PNUSAC000126	MN	jejuni	2015	Stool	ST45	DBRS16.0035	DBRK02.0164	SAMN04007135	SRR2218500
Non-1506UTDBR-1	PNUSAC021383	AK	jejuni	2015	Stool	ST45	DBRS16.2297	DBRK02.0757	SAMN21885749	SRR16102703
1509VTDBR-1	2015D-0143	VT	jejuni	2015	Stool	ST52	DBRS16.1615	DBRK02.1525	SAMN04147044	SRR2561784
1509VTDBR-1	2015D-0144	VT	jejuni	2015	Stool	ST52	DBRS16.1615	DBRK02.1525	SAMN04147045	SRR2561785
1509VTDBR-1	2015D-0145	VT	jejuni	2015	Stool	ST52	DBRS16.1615	DBRK02.1666	SAMN04147043	SRR2561783
1509VTDBR-1	2015D-0146	VT	jejuni	2015	Stool	ST52	DBRS16.1615	DBRK02.1666	SAMN04147046	SRR2561786
1509VTDBR-1	2015D-0147	VT	jejuni	2015	Stool	ST52	DBRS16.1615	DBRK02.1666	SAMN04147047	SRR2561787
1509VTDBR-1	2015D-0148	VT	jejuni	2015	Stool	ST52	DBRS16.1615	DBRK02.1666	SAMN04147042	SRR2561782
Non-1509VTDBR-1	CFSAN039433	TX	jejuni	2015	Stool	ST52	DBRS16.0060	NA	SAMN04148258	SRR2727656
Non-1509VTDBR-1	PNUSAC000209	MD	jejuni	2015	Stool	ST52	DBRS16.1615	DBRK02.1686	SAMN04386671	SRR3092138
Non-1509VTDBR-1	PNUSAC001409	WY	jejuni	2015	Stool	ST52	DBRS16.0060	DBRK02.1245	SAMN06248169	SRR5195477
Non-1509VTDBR-1	PNUSAC002098	WY	jejuni	2015	Stool	ST52	DBRS16.1142	DBRK02.1600	SAMN07205785	SRR5663534
Non-1509VTDBR-1	PNUSAC019716	AK	jejuni	2015	Stool	ST52	DBRS16.0872	DBRK02.1245	SAMN17131554	SRR13282474
1510WIDBR-1	PNUSAC000138	WI	jejuni	2015	Stool	ST8	DBRS16.0008	DBRK02.0148	SAMN04244353	SRR2916003
1510WIDBR-1	PNUSAC000139	WI	jejuni	2015	Stool	ST8	DBRS16.0008	DBRK02.0148	SAMN04244354	SRR2916004
1510WIDBR-1	PNUSAC000141	WI	jejuni	2015	Stool	ST8	DBRS16.0008	DBRK02.0148	SAMN04244356	SRR2916006
1510WIDBR-1	PNUSAC000142	WI	jejuni	2015	Stool	ST8	DBRS16.0008	DBRK02.0148	SAMN04244357	SRR2916007
1510WIDBR-1	PNUSAC000143	WI	jejuni	2015	Stool	ST8	DBRS16.0008	DBRK02.0148	SAMN04244358	SRR2916008
1510WIDBR-1	PNUSAC000144	WI	jejuni	2015	Stool	ST8	DBRS16.0008	DBRK02.0148	SAMN04244359	SRR2968333
1510WIDBR-1	PNUSAC000145	WI	jejuni	2015	Stool	ST8	DBRS16.0008	DBRK02.0148	SAMN04244360	SRR2916009
1510WIDBR-1	PNUSAC000147	WI	jejuni	2015	stool	ST8	DBRS16.0008	DBRK02.0148	SAMN04244361	SRR2916010
1510WIDBR-1	PNUSAC000149	WI	jejuni	2015	Stool	ST8	DBRS16.0008	DBRK02.0148	SAMN04244363	SRR2916012
1510WIDBR-1	PNUSAC000150	WI	jejuni	2015	stool	ST8	DBRS16.0008	DBRK02.0148	SAMN04244364	SRR2916013
Non-1510WIDBR-1	CFSAN038918	CA	jejuni	2015	Bovine Feces	ST8	DBRS16.0008	DBRK02.0148	SAMN04158379	SRR2968574
Non-1510WIDBR-1	FSIS1501343	AZ	jejuni	2015	Beef	ST8	DBRS16.0008	DBRK02.0148	SAMN04091204	SRR2423693
Non-1510WIDBR-1	PNUSAC000171	WI	jejuni	2015	Stool	ST8	DBRS16.0008	DBRK02.0148	SAMN04308884	SRR2970498
Non-1510WIDBR-1	PNUSAC019548	AK	jejuni	2015	Stool	ST8	DBRS16.0067	DBRK02.0148	SAMN17012104	SRR13204905

1602VTDBR-1	2016D-0045	VT	jejuni	2016	Stool	ST918	DBRS16.0048	DBRK02.1712	SAMN04579139	SRR3291011
1602VTDBR-1	2016D-0046	VT	jejuni	2016	Stool	ST918	DBRS16.0048	DBRK02.1712	SAMN04579140	SRR3291012
1602VTDBR-1	2016D-0047	VT	jejuni	2016	Stool	ST918	DBRS16.0048	DBRK02.1712	SAMN04579141	SRR3291013
1602VTDBR-1	2016D-0048	VT	jejuni	2016	Stool	ST918	DBRS16.0048	DBRK02.1712	SAMN04579142	SRR3291014
Non-1602VTDBR-1	FSIS1608657	IA	jejuni	2016	Chicken	ST918	DBRS16.0048	NA	SAMN06127113	SRR5091875
Non-1602VTDBR-1	PNUSAC000170	TX	jejuni	2015	Stool	ST918	DBRS16.3009	NA	SAMN04287726	SRR2954848
Non-1602VTDBR-1	PNUSAC000208	MD	jejuni	2015	Stool	ST918	DBRS16.0558	DBRS16.0558	SAMN04339737	SRR3029057
Non-1602VTDBR-1	PNUSAC000214	MD	jejuni	2015	Stool	ST918	DBRS16.0151	NA	SAMN04339742	SRR3029130
Non-1602VTDBR-1	PNUSAC001057	MD	jejuni	2016	Stool	ST918	DBRS16.0559	NA	SAMN06049087	SRR5222011
1607CADBR-1	PNUSAC000624	CA	jejuni	2016	Blood	ST8	DBRS16.0067	DBRK02.0148	SAMN05414057	SRR3930053
1607CADBR-1	PNUSAC000625	CA	jejuni	2016	Blood	ST8	DBRS16.0067	DBRK02.0148	SAMN05414058	SRR3930054
1607CADBR-1	PNUSAC000626	CA	jejuni	2016	Blood	ST8	DBRS16.0067	DBRK02.0148	SAMN05414059	SRR3930061
1607CADBR-1	PNUSAC000627	CA	jejuni	2016	Stool	ST8	DBRS16.0008	DBRK02.0148	SAMN05414060	SRR3930057
1607CADBR-1	PNUSAC000628	CA	jejuni	2016	Stool	ST21	DBRS16.0099	DBRK02.0210	SAMN05414061	SRR3930055
1607CADBR-1	PNUSAC000629	CA	jejuni	2016	Stool	ST21	DBRS16.0099	DBRK02.0210	SAMN05414062	SRR3930056
1607CADBR-1	PNUSAC000630	CA	jejuni	2016	Stool	ST806	DBRS16.1360	DBRK02.1786	SAMN05414063	SRR3930058
1607CADBR-1	PNUSAC000631	CA	jejuni	2016	Stool	ST806	DBRS16.1360	DBRK02.1786	SAMN05414064	SRR3930062
1607CADBR-1	PNUSAC000632	CA	jejuni	2016	Stool	ST6	Unrestricted	Unrestricted	SAMN05414065	SRR3930060
1607CADBR-1	PNUSAC000633	CA	jejuni	2016	Stool	ST432	DBRS16.0078	DBRK02.0199	SAMN05414066	SRR3930063
1607CADBR-1	PNUSAC000634	CA	jejuni	2016	Stool	ST8	DBRS16.0008	DBRK02.0148	SAMN05414067	SRR3930064
Non-1607CADBR-1	2016D-0168	VT	jejuni	2016	Stool	ST806	DBRS16.0185	Unrestricted	SAMN05504872	SRR3993655
Non-1607CADBR-1	PNUSAC000489	MD	jejuni	2016	Stool	ST8	DBRS16.0008	NA	SAMN05271591	SRR3710086
Non-1607CADBR-1	PNUSAC000534	NH	jejuni	2016	stool	ST8	DBRS16.0008	NA	SAMN05388838	SRR3922126
Non-1607CADBR-1	PNUSAC000553	UT	jejuni	2016	Stool	ST8	DBRS16.0008	NA	SAMN05323702	SRR3745099
Non-1607CADBR-1	PNUSAC000575	UT	jejuni	2016	Stool	ST8	DBRS16.0240	NA	SAMN05363718	SRR3758780
Non-1607CADBR-1	PNUSAC000584	UT	jejuni	2016	Stool	ST8	DBRS16.1860	NA	SAMN05363726	SRR3758941
Non-1607CADBR-1	PNUSAC000586	UT	jejuni	2016	Unknown	ST21	DBRS16.0206	NA	SAMN05382294	SRR3922129
Non-1607CADBR-1	PNUSAC000665	WY	jejuni	2016	Stool	ST8	DBRS16.0008	DBRK02.0148	SAMN05437764	SRR3969347
1609CODBR-1	PNUSAC000911	CO	jejuni	2016	Stool	ST8	DBRS16.0008	DBRK02.1272	SAMN05788710	SRR4280276
1609CODBR-1	PNUSAC000912	CO	jejuni	2016	Stool	ST8	DBRS16.0008	DBRK02.1272	SAMN05788711	SRR4280278
1609CODBR-1	PNUSAC000913	CO	jejuni	2016	Stool	ST8	DBRS16.0008	DBRK02.0028	SAMN05788712	SRR4280279
1609CODBR-1	PNUSAC000914	CO	jejuni	2016	Stool	ST8	DBRS16.0008	DBRK02.0028	SAMN05788713	SRR4280280
1609CODBR-1	PNUSAC000915	CO	jejuni	2016	Stool	ST8	DBRS16.0008	DBRK02.0028	SAMN05788714	SRR4280281
1609CODBR-1	PNUSAC000916	CO	jejuni	2016	Stool	ST8	DBRS16.0008	DBRK02.0028	SAMN05788715	SRR4280282
1609CODBR-1	PNUSAC000917	CO	jejuni	2016	Stool	ST8	DBRS16.0008	DBRK02.0028	SAMN05788716	SRR4280283
1609CODBR-1	PNUSAC000918	CO	jejuni	2016	Stool	ST8	DBRS16.0008	DBRK02.0028	SAMN05788717	SRR4280284
1609CODBR-1	PNUSAC000920	CO	jejuni	2016	Stool	ST8	DBRS16.0008	DBRK02.0028	SAMN05788718	SRR4280277
1609CODBR-1	PNUSAC000934	CO	jejuni	2016	Raw Milk	ST8	DBRS16.0008	DBRK02.0028	SAMN05833244	SRR4339886
1609CODBR-1	PNUSAC000953	CO	jejuni	2016	Stool	ST8	DBRS16.0008	DBRK02.0028	SAMN05894819	SRR4417838
1609CODBR-1	PNUSAC000957	CO	jejuni	2016	Raw Milk	ST8	DBRS16.0008	DBRK02.0028	SAMN05894833	SRR4417842
Non-1609CODBR-1	FSIS1608397	MO	jejuni	2016	Chicken	ST8	DBRS16.0008	NA	SAMN06046117	SRR5043978
Non-1609CODBR-1	PNUSAC000649	MN	jejuni	2016	Stool	ST8	DBRS16.0060	DBRK02.0482	SAMN05468403	SRR3993650
Non-1609CODBR-1	PNUSAC000792	UT	jejuni	2016	Stool	ST8	DBRS16.0008	NA	SAMN05615914	SRR4106546
Non-1609CODBR-1	PNUSAC000900	NH	jejuni	2016	stool	ST8	DBRS16.0008	NA	SAMN05804543	SRR4292042
Non-1609CODBR-1	PNUSAC000924	WY	jejuni	2016	Stool	ST8	DBRS16.1766	DBRK02.1823	SAMN05819032	SRR4297644
Non-1609CODBR-1	PNUSAC001052	ND	jejuni	2016	Stool	ST8	NA	NA	SAMN06064407	SRR5060305
Non-1609CODBR-1	PNUSAC001125	UT	jejuni	2016	Stool	ST8	DBRS16.0008	NA	SAMN06050847	SRR5508065
Non-1609CODBR-1	PNUSAC001126	UT	jejuni	2016	Stool	ST8	DBRS16.0008	NA	SAMN06050848	SRR5508068
1612OHDBR-1	B16-25748	OH	jejuni	2016	Raw Milk	ST1244	DBRS16.0575	DBRK02.0686	SAMN06068091	SRR5076583
1612OHDBR-1	PNUSAC001092	OH	jejuni	2016	Blood	ST1244	DBRS16.0575	DBRK02.0686	SAMN06043894	SRR5045308
1612OHDBR-1	PNUSAC001218	OH	jejuni	2016	Stool	ST1244	DBRS16.0575	DBRK02.0686	SAMN06124519	SRR5097313
Non-1612OHDBR-1	PNUSAC001001	MD	jejuni	2016	Stool	ST1244	DBRS16.0006	NA	SAMN06117676	SRR5097301
Non-1612OHDBR-1	PNUSAC001386	UT	jejuni	2016	Stool	ST1244	DBRS16.3410	NA	SAMN06231014	SRR5178023
Non-1612OHDBR-1	PNUSAC001414	MD	jejuni	2016	Stool	ST1244	DBRS16.0006	NA	SAMN06285319	SRR5230115
1707MADBR-1	2017D-0142	VT	jejuni	2017	Stool	ST6647	DBRS16.0164	DBRK02.1088	SAMN07615389	SRR6014984
1707MADBR-1	2017D-0143	VT	jejuni	2017	Stool	ST6647	DBRS16.0164	DBRK02.1934	SAMN07615388	SRR6014980
1707MADBR-1	2017D-0144	VT	jejuni	2017	Stool	ST6647	DBRS16.0164	DBRK02.1934	SAMN07615387	SRR6014982
1707MADBR-1	PNUSAC002399	MA	jejuni	2017	Stool	ST6647	DBRS16.0164	DBRK02.1088	SAMN07451849	SRR5918210
1707MADBR-1	PNUSAC002400	MA	jejuni	2017	Stool	ST6647	DBRS16.0164	DBRK02.1088	SAMN07451850	SRR5918206
1707MADBR-1	PNUSAC002401	MA	jejuni	2017	Stool	ST6647	DBRS16.0164	DBRK02.1088	SAMN07451851	SRR5918208
1707MADBR-1	PNUSAC002402	MA	jejuni	2017	Stool	ST6647	DBRS16.0164	DBRK02.1088	SAMN07451852	SRR5918218
Non-1707MADBR-1	PNUSAC002234	MD	jejuni	2017	Stool	ST6647	DBRS16.0164	NA	SAMN07358264	SRR5838540
Non-1707MADBR-1	PNUSAC006471	SC	jejuni	2017	Stool	ST6647	NA	NA	SAMN10221739	SRR7984977
Non-1707MADBR-1	PNUSAC007205	CT	jejuni	2017	Stool	ST6647	NA	NA	SAMN10589336	SRR8306551

1708FLDBR-1	2016AY-0371	GA	jejuni	2016	Stool	ST2109	NA	NA	SAMN08098203	SRR6354019
1708FLDBR-1	2016AY-1256	CT	jejuni	2016	Stool	ST2109	NA	NA	SAMN08098204	SRR6354026
1708FLDBR-1	2017D-0132	MO	jejuni	2017	Stool	ST2109	DBRS16.3339	Unrestricted	SAMN07615386	SRR6014503
1708FLDBR-1	2017D-0136	FL	jejuni	2017	Canine Feces	ST2109	DBRS16.3648	Unrestricted	SAMN07504897	SRR6171586
1708FLDBR-1	2017D-0138	FL	jejuni	2017	Canine Feces	ST2109	DBRS16.3339	Unrestricted	SAMN07504895	SRR6171584
1708FLDBR-1	2017D-0139	FL	jejuni	2017	Canine Feces	ST2109	DBRS16.3648	Unrestricted	SAMN07504896	SRR6171587
1708FLDBR-1	2017D-0140	FL	jejuni	2017	Canine Feces	ST2109	DBRS16.3339	Unrestricted	SAMN07504898	SRR6171583
1708FLDBR-1	2017D-0163	FL	jejuni	2017	Canine Feces	ST2109	DBRS16.0170	Unrestricted	SAMN07674670	SRR6171585
1708FLDBR-1	2017D-0180	TN	jejuni	2017	Stool	ST2109	DBRS16.0170	DBRK02.1949	SAMN07605519	SRR6202903
1708FLDBR-1	CFSAN069649	OH	jejuni	2017	Canine Feces	ST2109	DBRS16.3648	Unrestricted	SAMN07710399	SRR6080585
1708FLDBR-1	CFSAN071607	OH	jejuni	2017	Canine Feces	ST2109	DBRS16.3339	Unrestricted	SAMN08025804	SRR6297976
1708FLDBR-1	CFSAN076605	FL	jejuni	2018	Canine Feces	ST2109	DBRS16.3648	NA	SAMN08624512	SRR6814032
1708FLDBR-1	CFSAN076609	FL	jejuni	2018	Canine Feces	ST2109	DBRS16.0170	NA	SAMN08624509	SRR6813854
1708FLDBR-1	CFSAN076610	FL	jejuni	2018	Canine Feces	ST2109	DBRS16.3648	NA	SAMN08624507	SRR6814049
1708FLDBR-1	PNUSAC000260	WY	jejuni	2016	Stool	ST2109	DBRS16.1390	Unrestricted	SAMN05255135	SRR3710099
1708FLDBR-1	PNUSAC000392	MD	jejuni	2016	Stool	ST2109	DBRS16.0170	NA	SAMN04961828	SRR3492160
1708FLDBR-1	PNUSAC000643	MD	jejuni	2016	Stool	ST2109	DBRS16.0164	NA	SAMN05413992	SRR3932384
1708FLDBR-1	PNUSAC000960	WY	jejuni	2016	Stool	ST2109	DBRS16.0170	Unrestricted	SAMN05908962	SRR4427165
1708FLDBR-1	PNUSAC001027	NH	jejuni	2016	Stool	ST2109	DBRS16.3339	NA	SAMN06007277	SRR5060317
1708FLDBR-1	PNUSAC001091	WY	jejuni	2016	Stool	ST2109	DBRS16.0170	Unrestricted	SAMN06036245	SRR5039804
1708FLDBR-1	PNUSAC001449	UT	jejuni	2016	Stool	ST2109	DBRS16.3339	Unrestricted	SAMN06562215	SRR5337178
1708FLDBR-1	PNUSAC001462	MD	jejuni	2016	Stool	ST2109	DBRS16.0170	NA	SAMN06285341	SRR5230136
1708FLDBR-1	PNUSAC001641	CT	jejuni	2017	Stool	ST2109	NA	NA	SAMN06644938	SRR5382106
1708FLDBR-1	PNUSAC001644	CT	jejuni	2017	Stool	ST2109	NA	NA	SAMN06644941	SRR5382101
1708FLDBR-1	PNUSAC001816	OH	jejuni	2017	Stool	ST2109	DBRS16.0170	Unrestricted	SAMN06759833	SRR5456705
1708FLDBR-1	PNUSAC001817	OH	jejuni	2017	Stool	ST2109	DBRS16.0170	Unrestricted	SAMN06759834	SRR5456706
1708FLDBR-1	PNUSAC001842	UT	jejuni	2017	Stool	ST2109	DBRS16.0170	NA	SAMN07135004	SRR5581839
1708FLDBR-1	PNUSAC001921	MD	jejuni	2017	Stool	ST2109	DBRS16.0170	NA	SAMN07124712	SRR5581935
1708FLDBR-1	PNUSAC002416	OH	jejuni	2017	Stool	ST2109	DBRS16.3583	Unrestricted	SAMN07629386	SRR6048525
1708FLDBR-1	PNUSAC002518	MD	jejuni	2017	Stool	ST2109	DBRS16.3339	Unrestricted	SAMN07510040	SRR5937917
1708FLDBR-1	PNUSAC002574	FL	jejuni	2017	Canine Feces	ST2109	DBRS16.3648	Unrestricted	SAMN07504894	SRR5935033
1708FLDBR-1	PNUSAC002580	FL	jejuni	2017	Canine Feces	ST2109	DBRS16.3339	Unrestricted	SAMN07504900	SRR5935048
1708FLDBR-1	PNUSAC002743	WI	jejuni	2017	Stool	ST2109	DBRS16.0170	DBRK02.0488	SAMN07646583	SRR6048555
1708FLDBR-1	PNUSAC002744	WI	jejuni	2017	Stool	ST2109	DBRS16.0170	Unrestricted	SAMN07646584	SRR6048556
1708FLDBR-1	PNUSAC002880	NH	jejuni	2017	Stool	ST2109	DBRS16.0170	NA	SAMN07761796	SRR6152533
1708FLDBR-1	PNUSAC002906	KS	jejuni	2017	Stool	ST2109	DBRS16.3648	NA	SAMN07790191	SRR6179762
1708FLDBR-1	PNUSAC003024	WI	jejuni	2016	Stool	ST2109	DBRS16.0164	DBRK02.0488	SAMN07837859	SRR6232441
1708FLDBR-1	PNUSAC003026	WI	jejuni	2016	Stool	ST2109	DBRS16.0164	NA	SAMN07837861	SRR6232434
1708FLDBR-1	PNUSAC003029	WI	jejuni	2017	Stool	ST2109	DBRS16.0164	NA	SAMN07837874	SRR6232448
1708FLDBR-1	PNUSAC003051	WI	jejuni	2016	Stool	ST2109	DBRS16.0164	NA	SAMN07837858	SRR6232430
1708FLDBR-1	PNUSAC003081	MA	jejuni	2017	Stool	ST2109	DBRS16.0164	Unrestricted	SAMN07988994	SRR6313161
1708FLDBR-1	PNUSAC003089	OH	jejuni	2016	Stool	ST2109	DBRS16.0170	DBRK02.0488	SAMN07977045	SRR6261379
1708FLDBR-1	PNUSAC003152	OH	jejuni	2017	Stool	ST2109	DBRS16.0170	Unrestricted	SAMN08014318	SRR6289219
1708FLDBR-1	PNUSAC003153	OH	jejuni	2017	Stool	ST2109	DBRS16.3339	Unrestricted	SAMN08014319	SRR6289213
1708FLDBR-1	PNUSAC003169	MA	jejuni	2017	Stool	ST2109	DBRS16.3339	Unrestricted	SAMN08016191	SRR6289218
1708FLDBR-1	PNUSAC003197	WI	jejuni	2017	Stool	ST2109	DBRS16.0164	DBRK02.0488	SAMN08050098	SRR6315881
1708FLDBR-1	PNUSAC003228	UT	jejuni	2017	Stool	ST2109	DBRS16.3339	NA	SAMN08135041	SRR6353935
1708FLDBR-1	PNUSAC003268	OH	jejuni	2017	Stool	ST2109	DBRS16.0170	DBRK02.0488	SAMN08113727	SRR6337383
1708FLDBR-1	PNUSAC003286	WI	jejuni	2017	Stool	ST2109	DBRS16.0164	DBRK02.0488	SAMN08115708	SRR6337362
1708FLDBR-1	PNUSAC003292	NY	jejuni	2017	Stool	ST2109	DBRS16.0170	Unrestricted	SAMN08130249	SRR6353934
1708FLDBR-1	PNUSAC003325	FL	jejuni	2017	Stool	ST2109	NA	NA	SAMN08114840	SRR6337345
1708FLDBR-1	PNUSAC003513	WI	jejuni	2017	Canine Feces	ST2109	DBRS16.0164	DBRK02.0488	SAMN08240012	SRR6424098
1708FLDBR-1	PNUSAC003525	KS	jejuni	2017	Stool	ST2109	DBRS16.3697	Unrestricted	SAMN08285080	SRR6439804
1708FLDBR-1	PNUSAC003734	UT	jejuni	2018	Stool	ST2109	DBRS16.0164	NA	SAMN08513612	SRR6703680
1708FLDBR-1	PNUSAC003753	GA	jejuni	2018	Stool	ST2109	NA	NA	SAMN08556545	SRR6741489
1708FLDBR-1	PNUSAC003754	GA	jejuni	2018	Canine Feces	ST2109	NA	NA	SAMN08556540	SRR6741488
1708FLDBR-1	PNUSAC003758	IL	jejuni	2017	Stool	ST2109	NA	NA	SAMN08517331	SRR6706105
1708FLDBR-1	PNUSAC003810	WI	jejuni	2018	Stool	ST2109	DBRS16.0164	DBRK02.0488	SAMN08615568	SRR6785781
1708FLDBR-1	PNUSAC003814	OH	jejuni	2018	Stool	ST2109	DBRS16.3648	Unrestricted	SAMN08729633	SRR6856517
1708FLDBR-1	PNUSAC003829	FL	jejuni	2018	Stool	ST2109	NA	NA	SAMN08633868	SRR6801479
1708FLDBR-1	PNUSAC003858	TN	jejuni	2018	Stool	ST2109	NA	NA	SAMN08849554	SRR6929981
1708FLDBR-1	PNUSAC003859	TN	jejuni	2018	Stool	ST2109	NA	NA	SAMN08849550	SRR6929982
Non-1708FLDBR-1	PNUSAC002077	UT	jejuni	2017	Stool	ST2109	DBRS16.0063	NA	SAMN07249899	SRR5710040
Non-1708FLDBR-1	PNUSAC002131	TX	jejuni	2017	Stool	ST2109	DBRS16.1176	NA	SAMN07249750	SRR5710201
Non-1708FLDBR-1	PNUSAC002999	MD	jejuni	2017	Stool	ST2109	DBRS16.0170	NA	SAMN08007248	SRR6282658
Non-1708FLDBR-1	PNUSAC003464	MN	jejuni	2017	Stool	ST2109	DBRS16.0336	DBRK02.2024	SAMN08275909	SRR6445056
1802VADBR-1	PNUSAC003735	VA	jejuni	2018	Stool	ST50	DBRS16.1094	DBRK02.1979	SAMN08556544	SRR6741487
1802VADBR-1	PNUSAC003736	VA	jejuni	2018	Stool	ST50	DBRS16.1094	DBRK02.1979	SAMN08556543	SRR6741492
1802VADBR-1	PNUSAC003738	VA	jejuni	2018	Stool	ST50	DBRS16.1094	DBRK02.1979	SAMN08556541	SRR6741491
Non-1802VADBR-1	2018AY-0523	NY	jejuni	2018	Stool	ST50	NA	NA	SAMN17799307	SRR13633256
Non-1802VADBR-1	PNUSAC003568	UT	jejuni	2017	Stool	ST50	DBRS16.0450	NA	SAMN08355211	SRR6467807
Non-1802VADBR-1	PNUSAC004114	MD	jejuni	2018	Stool	ST50	DBRS16.0404	NA	SAMN08941563	SRR7013034
Non-1802VADBR-1	PNUSAC004144	TN	jejuni	2018	Stool	ST50	NA	NA	SAMN08935178	SRR7007262
Non-1802VADBR-1	PNUSAC005436	NM	jejuni	2018	Stool	ST50	NA	NA	SAMN09714401	SRR7590078
Non-1802VADBR-1	PNUSAC006496	SC	jejuni	2018	Stool	ST50	NA	NA	SAMN10287197	SRR8103776
Non-1802VADBR-1	PNUSAC006498	SC	jejuni	2018	Stool	ST50	NA	NA	SAMN10287199	SRR8103401
Non-1802VADBR-1	PNUSAC007324	MN	jejuni	2018	Stool	ST50	NA	NA	SAMN10669096	SRR8382729
Non-1802VADBR-1	PNUSAC007327	MN	jejuni	2018	Stool	ST50	NA	NA	SAMN10669093	SRR8382714

1906NVDBR-1	PNUSAC008091	MN	jejuni	2019	Stool	ST2109	NA	NA	SAMN11022492	SRR8632918
1906NVDBR-1	PNUSAC008467	OH	jejuni	2019	Stool	ST2109	NA	NA	SAMN11109455	SRR8715354
1906NVDBR-1	PNUSAC008908	OH	jejuni	2019	Stool	ST2109	NA	NA	SAMN11439804	SRR8914616
1906NVDBR-1	PNUSAC009087	OH	jejuni	2019	Stool	ST2109	NA	NA	SAMN11490887	SRR8950402
1906NVDBR-1	PNUSAC009268	MN	jejuni	2019	Stool	ST2109	NA	NA	SAMN14396413	SRR11342509
1906NVDBR-1	PNUSAC009270	GA	jejuni	2019	Stool	ST5453	NA	NA	SAMN11582981	SRR9019622
1906NVDBR-1	PNUSAC009357	MN	jejuni	2019	Stool	ST5453	NA	NA	SAMN14396415	SRR11342510
1906NVDBR-1	PNUSAC009606	FL	jejuni	2019	Stool	ST2109	NA	NA	SAMN14390643	SRR11321380
1906NVDBR-1	PNUSAC009659	WY	jejuni	2019	Stool	ST2109	NA	NA	SAMN14608373	SRR11557474
1906NVDBR-1	PNUSAC009690	MN	jejuni	2019	Stool	ST5453	NA	NA	SAMN14396414	SRR11342520
1906NVDBR-1	PNUSAC009836	NV	jejuni	2019	Stool	ST5453	NA	NA	SAMN12108601	SRR9335747
1906NVDBR-1	PNUSAC009838	NV	jejuni	2019	Stool	ST2109	NA	NA	SAMN12108597	SRR9335745
1906NVDBR-1	PNUSAC009898	NV	jejuni	2019	Stool	ST2109	NA	NA	SAMN12307459	SRR9705411
1906NVDBR-1	PNUSAC009948	SC	jejuni	2019	Stool	ST5453	NA	NA	SAMN12127688	SRR9593495
1906NVDBR-1	PNUSAC010116	OH	jejuni	2019	Stool	ST2109	NA	NA	SAMN12421851	SRR9879560
1906NVDBR-1	PNUSAC010326	TN	jejuni	2019	Stool	ST2109	NA	NA	SAMN12528564	SRR9921270
1906NVDBR-1	PNUSAC010799	MD	jejuni	2019	Stool	ST5453	NA	NA	SAMN12504884	SRR9901717
1906NVDBR-1	PNUSAC010840	MN	jejuni	2019	Stool	ST2109	NA	NA	SAMN12518212	SRR9915954
1906NVDBR-1	PNUSAC011331	KY	jejuni	2019	Stool	ST2109	NA	NA	SAMN12642726	SRR10024862
1906NVDBR-1	PNUSAC011593	MN	jejuni	2019	Stool	ST5453	NA	NA	SAMN12736016	SRR10103059
1906NVDBR-1	PNUSAC011649	KY	jejuni	2019	Stool	ST5453	NA	NA	SAMN12745436	SRR10116195
1906NVDBR-1	PNUSAC011680	OH	jejuni	2019	Stool	ST2109	NA	NA	SAMN12766991	SRR10124058
1906NVDBR-1	PNUSAC011781	SC	jejuni	2019	Stool	ST2109	NA	NA	SAMN12830807	SRR10174197
1906NVDBR-1	PNUSAC012595	UT	jejuni	2019	Stool	ST2109	NA	NA	SAMN13032462	SRR10281749
1906NVDBR-1	PNUSAC012739	CT	jejuni	2019	Stool	ST2109	NA	NA	SAMN13058937	SRR10311572
1906NVDBR-1	PNUSAC012750	NV	jejuni	2019	Stool	ST2109	NA	NA	SAMN13058811	SRR10310359
1906NVDBR-1	PNUSAC012931	UT	jejuni	2019	Stool	ST2109	NA	NA	SAMN13116031	SRR10348600
1906NVDBR-1	PNUSAC012960	MN	jejuni	2019	Stool	ST5453	NA	NA	SAMN13116628	SRR10348703
1906NVDBR-1	PNUSAC013773	UT	jejuni	2019	Stool	ST5453	NA	NA	SAMN13392908	SRR10536684
1906NVDBR-1	PNUSAC013845	IL	jejuni	2019	Stool	ST5453	NA	NA	SAMN13473218	SRR10579508
1906NVDBR-1	PNUSAC014266	MN	jejuni	2019	Stool	ST5453	NA	NA	SAMN13656684	SRR10747831
1906NVDBR-1	PNUSAC014574	CT	jejuni	2019	Stool	ST2109	NA	NA	SAMN13699046	SRR10803555
1906NVDBR-1	PNUSAC014904	MI	jejuni	2019	Stool	ST5453	NA	NA	SAMN14049758	SRR11032272
1906NVDBR-1	PNUSAC015167	NV	jejuni	2020	Stool	ST2109	NA	NA	SAMN13897151	SRR10919697
1906NVDBR-1	PNUSAC015390	MN	jejuni	2020	Stool	ST5453	NA	NA	SAMN13975226	SRR11004666
1906NVDBR-1	PNUSAC015391	WI	jejuni	2020	Stool	ST5453	NA	NA	SAMN13975217	SRR11004664
1906NVDBR-1	PNUSAC015415	NV	jejuni	2020	Stool	ST2109	NA	NA	SAMN13977299	SRR11007929
1906NVDBR-1	PNUSAC015699	MN	jejuni	2020	Stool	ST2109	NA	NA	SAMN14088458	SRR11073224
1906NVDBR-1	PNUSAC015725	IA	jejuni	2020	Canine Feces	ST5453	NA	NA	SAMN14124791	SRR11100741
1906NVDBR-1	PNUSAC015726	IA	jejuni	2020	Stool	ST5453	NA	NA	SAMN14124790	SRR11100742
1906NVDBR-1	PNUSAC015802	MN	jejuni	2020	Canine Feces	ST5453	NA	NA	SAMN14169401	SRR11151282
1906NVDBR-1	PNUSAC015969	IA	jejuni	2020	Stool	ST5453	NA	NA	SAMN14267089	SRR11218506
1906NVDBR-1	PNUSAC016044	MN	jejuni	2020	Stool	ST5453	NA	NA	SAMN14332977	SRR11268064
1906NVDBR-1	PNUSAC016201	UT	jejuni	2020	Stool	ST2109	NA	NA	SAMN14437785	SRR11401885
1906NVDBR-1	PNUSAC016239	MN	jejuni	2020	Stool	ST2109	NA	NA	SAMN14534258	SRR11477434
1906NVDBR-1	PNUSAC016454	NV	jejuni	2020	Stool	ST5453	NA	NA	SAMN14841193	SRR11700244
1906NVDBR-1	PNUSAC016705	AR	jejuni	2020	Stool	ST5453	NA	NA	SAMN15102194	SRR11925978
1906NVDBR-1	PNUSAC016719	MN	jejuni	2020	Stool	ST2109	NA	NA	SAMN15147191	SRR11935439
1906NVDBR-1	PNUSAC016786	CT	jejuni	2020	Stool	ST2109	NA	NA	SAMN15186874	SRR11965729
1906NVDBR-1	PNUSAC017003	UT	jejuni	2020	Stool	ST2109	NA	NA	SAMN15390165	SRR12101074
1906NVDBR-1	PNUSAC017331	MN	jejuni	2020	Stool	ST2109	NA	NA	SAMN15580008	SRR12265851
1906NVDBR-1	PNUSAC017523	MN	jejuni	2020	Stool	ST2109	NA	NA	SAMN15671853	SRR12346709
1906NVDBR-1	PNUSAC017543	MN	jejuni	2020	Stool	ST2109	NA	NA	SAMN15690443	SRR12362515
1906NVDBR-1	PNUSAC018065	NV	jejuni	2020	Stool	ST5453	NA	NA	SAMN16090094	SRR12618383
1906NVDBR-1	PNUSAC018549	MD	jejuni	2020	Stool	ST5453	NA	NA	SAMN16428846	SRR12817823
1906NVDBR-1	PNUSAC019851	MN	jejuni	2020	Stool	ST5453	NA	NA	SAMN17310596	SRR13430963
1906NVDBR-1	PNUSAC019935	CT	jejuni	2021	Stool	ST2109	NA	NA	SAMN17577705	SRR13532117
Non-1906NVDBR-1	PNUSAC010005	GA	jejuni	2019	Stool	ST2109	NA	NA	SAMN12262705	SRR9675986
Non-1906NVDBR-1	PNUSAC010739	SD	jejuni	2019	Stool	ST2109	NA	NA	SAMN12497280	SRR9901376
Non-1906NVDBR-1	PNUSAC013324	NM	jejuni	2019	Stool	ST2109	NA	NA	SAMN13284289	SRR10442267
Non-1906NVDBR-1	PNUSAC013325	NM	jejuni	2019	Stool	ST2109	NA	NA	SAMN13284297	SRR10442265
Non-1906NVDBR-1	PNUSAC015558	NM	jejuni	2019	Stool	ST2109	NA	NA	SAMN14056893	SRR11038182
Non-1906NVDBR-1	PNUSAC017469	SC	jejuni	2020	Stool	ST5453	NA	NA	SAMN15638730	SRR12316524
Non-1906NVDBR-1	PNUSAC017672	MN	jejuni	2020	Stool	ST5453	NA	NA	SAMN15737988	SRR12393623
Non-1906NVDBR-1	PNUSAC020352	MN	jejuni	2021	Stool	ST5453	NA	NA	SAMN17914259	SRR13708323
1907VTDBR-1	PNUSAC009682	VT	jejuni	2019	Stool	ST4559	NA	NA	SAMN15521824	SRR12212833
1907VTDBR-1	PNUSAC010066	VT	jejuni	2019	Stool	ST4559	NA	NA	SAMN15521818	SRR12212840
1907VTDBR-1	PNUSAC010069	VT	jejuni	2019	Stool	ST4559	NA	NA	SAMN15521826	SRR12212838
1907VTDBR-1	PNUSAC010072	VT	jejuni	2019	Stool	ST4559	NA	NA	SAMN15521823	SRR12212841
1907VTDBR-1	PNUSAC010073	VT	jejuni	2019	Stool	ST4559	NA	NA	SAMN16428353	SRR12817698
Non-1907VTDBR-1	FSIS12031892	ME	jejuni	2020	Beef	ST4559	NA	NA	SAMN15594311	SRR12282301
Non-1907VTDBR-1	FSIS22028273	VA	jejuni	2020	Chicken	ST4559	NA	NA	SAMN15226653	SRR12005812
Non-1907VTDBR-1	PNUSAC013040	WY	jejuni	2007	Unknown	ST4559	NA	NA	SAMN14605729	SRR11554688
2102NHDBR-1	PNUSAC020370	MA	jejuni	2021	Stool	ST8	NA	NA	SAMN17924390	SRR13719211
2102NHDBR-1	PNUSAC020468	NH	jejuni	2021	Stool	ST8	NA	NA	SAMN18046987	SRR13776834
2102NHDBR-1	PNUSAC020469	NH	jejuni	2021	Stool	ST8	NA	NA	SAMN18046986	SRR13776827
2102NHDBR-1	PNUSAC020470	NH	jejuni	2021	Stool	ST8	NA	NA	SAMN18046984	SRR13776830
2102NHDBR-1	PNUSAC020471	NH	jejuni	2021	Stool	ST8	NA	NA	SAMN18046983	SRR13776826
2102NHDBR-1	PNUSAC020472	ME	jejuni	2021	Stool	ST8	NA	NA	SAMN18046981	SRR13776833
2102NHDBR-1	PNUSAC020473	ME	jejuni	2021	Stool	ST8	NA	NA	SAMN18046979	SRR13776832
2102NHDBR-1	PNUSAC020475	ME	jejuni	2021	Stool	ST8	NA	NA	SAMN18046976	SRR13776829
2102NHDBR-1	PNUSAC020476	NH	jejuni	2021	Raw Milk	ST8	NA	NA	SAMN18050330	SRR13777142
2102NHDBR-1	PNUSAC020490	ME	jejuni	2021	Stool	ST8	NA	NA	SAMN18095806	SRR13815804
2102NHDBR-1	PNUSAC020491	ME	jejuni	2021	Stool	ST8	NA	NA	SAMN18095804	SRR13815805
Non-2102NHDBR-1	PNUSAC020623	VT	jejuni	2021	Stool	ST8	NA	NA	SAMN18351021	SRR13998349
Non-2102NHDBR-1	PNUSAC020837	NM	jejuni	2021	Blood	ST8	NA	NA	SAMN18743841	SRR14240508
Non-2102NHDBR-1	PNUSAC020915	MN	jejuni	2021	Stool	ST8	NA	NA	SAMN18796889	SRR14274510

NA indicates that PFGE was not performed

Non- before outbreak code indicates PFGE and/or 7-gene MLST matching sporadic isolates.

Supplemental Table 2. The median Baker's Gamma Correlation Coefficient and Cophenetic Correlation Coefficient comparing cgMLST, wgMLST, and hqSNP analysis for all *Campylobacter* outbreaks in this study.

Outbreak	Species	Workflows Compared	Baker's Gamma Correlation Coefficient	Cophenetic Correlation Coefficient
0810PADBR-1	jejuni	cgMLST and wgMLST	0.9860092	0.9993735
0810PADBR-1	jejuni	cgMLST and hqSNP	0.7191145	0.9983805
0810PADBR-1	jejuni	wgMLST and hqSNP	0.7051005	0.9957472
1008MTDBR-1	jejuni	cgMLST and wgMLST	1.0000000	0.9998151
1008MTDBR-1	jejuni	cgMLST and hqSNP	0.6304844	0.9639828
1008MTDBR-1	jejuni	wgMLST and hqSNP	0.6304844	0.9586905
1302AKDBB-1	coli	cgMLST and wgMLST	0.9812894	0.9965703
1302AKDBB-1	coli	cgMLST and hqSNP	0.8130939	0.9332807
1302AKDBB-1	coli	wgMLST and hqSNP	0.8077682	0.9310140
1506UTDBR-1	jejuni	cgMLST and wgMLST	0.9959603	0.9997282
1506UTDBR-1	jejuni	cgMLST and hqSNP	0.9297008	0.8643298
1506UTDBR-1	jejuni	wgMLST and hqSNP	0.9316800	0.8596433
1509VTDBR-1	jejuni	cgMLST and wgMLST	0.9909596	0.9913325
1509VTDBR-1	jejuni	cgMLST and hqSNP	0.9703247	0.9761045
1509VTDBR-1	jejuni	wgMLST and hqSNP	0.9815400	0.9943429
1510WIDBR-1	jejuni	cgMLST and wgMLST	0.9989253	0.9997394
1510WIDBR-1	jejuni	cgMLST and hqSNP	0.9781050	0.9933422
1510WIDBR-1	jejuni	wgMLST and hqSNP	0.9770302	0.9922536
1602VTDBR-1	jejuni	cgMLST and wgMLST	0.9993348	0.9997551
1602VTDBR-1	jejuni	cgMLST and hqSNP	0.9889874	0.9856474
1602VTDBR-1	jejuni	wgMLST and hqSNP	0.9896526	0.9849984
1607CADBR-1	jejuni	cgMLST and wgMLST	0.9998785	0.9998092
1607CADBR-1	jejuni	cgMLST and hqSNP	0.9526931	0.8178409
1607CADBR-1	jejuni	wgMLST and hqSNP	0.9527694	0.8278065
1609CODBR-1	jejuni	cgMLST and wgMLST	0.9651204	0.9922949
1609CODBR-1	jejuni	wgMLST and hqSNP	0.9377111	0.9806019
1609CODBR-1	jejuni	cgMLST and hqSNP	0.9295532	0.9856743
1612OHDBR-1	jejuni	cgMLST and wgMLST	1.0000000	0.9996135
1612OHDBR-1	jejuni	cgMLST and hqSNP	0.9904459	0.9974738
1612OHDBR-1	jejuni	wgMLST and hqSNP	0.9904459	0.9990356
1707MADBR-1	jejuni	cgMLST and wgMLST	1.0000000	0.9989644
1707MADBR-1	jejuni	wgMLST and hqSNP	0.9506006	0.9846651
1707MADBR-1	jejuni	cgMLST and hqSNP	0.9506006	0.9845855
1708FLDBR-1a	jejuni	cgMLST and wgMLST	0.9818695	0.9995117
1708FLDBR-1a	jejuni	cgMLST and hqSNP	0.9607865	0.9943448
1708FLDBR-1a	jejuni	wgMLST and hqSNP	0.9548457	0.9949367
1708FLDBR-1b	jejuni	cgMLST and wgMLST	0.9652292	0.9997944
1708FLDBR-1b	jejuni	cgMLST and hqSNP	0.7934984	0.9907869
1708FLDBR-1b	jejuni	wgMLST and hqSNP	0.7962167	0.9927127
1802VADBR-1	jejuni	cgMLST and wgMLST	0.9989737	0.9988478
1802VADBR-1	jejuni	cgMLST and hqSNP	0.9989737	0.9681096
1802VADBR-1	jejuni	wgMLST and hqSNP	1.0000000	0.9665958
1906NVDBR-1	jejuni	cgMLST and wgMLST	0.9986404	0.9999761
1906NVDBR-1	jejuni	cgMLST and hqSNP	0.9809512	0.9931390
1906NVDBR-1	jejuni	wgMLST and hqSNP	0.9806910	0.9935987
1907VTDBR-1	jejuni	cgMLST and wgMLST	0.9895125	0.9999971
1907VTDBR-1	jejuni	cgMLST and hqSNP	0.9037698	0.9962365
1907VTDBR-1	jejuni	wgMLST and hqSNP	0.9142574	0.9963307
2102OHDBR-1	jejuni	cgMLST and wgMLST	0.9762348	0.9993595
2102OHDBR-1	jejuni	cgMLST and hqSNP	0.7641990	0.9966430
2102OHDBR-1	jejuni	wgMLST and hqSNP	0.7752498	0.9963568
